# The Metamorphosis. The impact of a young family member’s problematic substance use on family life: a meta-ethnography

**DOI:** 10.1080/17482631.2023.2202970

**Published:** 2023-04-20

**Authors:** Sari Kaarina Lindeman, Lennart Lorås, Kristine Berg Titlestad, Terese Bondas

**Affiliations:** a Faculty of Health and Social Sciences, Western Norway University of Applied Sciences, Bergen, Norway; b Faculty of Health Sciences, University of Stavanger, Stavanger, Norway

**Keywords:** Adolescence, family impact, family life, meta-ethnography, problematic substance use, young adults

## Abstract

**Purpose:**

This meta-ethnography seeks to provide insight into the impact that a young family member’s problematic substance use has on family life.

**Background:**

Problematic substance use (PSU) usually emerges in adolescence or young adulthood. Living with a family member with PSU is highly stressful. An understanding is needed of families’ experiences and their needs for adapted help and support, hence we have explored the impact of a young family member’s PSU on family life.

**Methods:**

Systematic literature searches for qualitative research that explores the impact of PSU on family life and family relationships were conducted and the seven stages of meta-ethnography were used.

**Results:**

Fifteen articles were included. The Metamorphosis was established as an overarching metaphor. Five main themes accompany this metaphor: *stranger in the family; injuring chaos; no trust any more; family lock-up; and helpless societies*.

**Conclusion:**

The Metamorphosis reflects the all-embracing change experienced by families. Family members have felt powerless and helpless; often they wish to stay involved but do not know how. PSU at a young age can develop into lifelong chronic health challenges. Family-oriented help must be readily available in this phase as parents and siblings become deeply involved. Family involvement is seldom incorporated into routine treatment practices; such incorporation is therefore needed.

## Introduction

Problematic substance use (PSU) usually emerges in adolescence or young adulthood. Most research suggests that adolescence (the period between the ages of 12 and 17) is a critical risk period for the initiation of substance use and that substance use may peak among young people aged 18 to 25 (World Drug Report, 2018). In addition, the legal substance alcohol is used by more than a quarter of all those aged 15 to 19 worldwide (WHO, 2018). PSU (e.g., alcohol and/or drug use) is a serious health problem associated with a severe threat of premature death (WHO, 2018). PSU at any age is difficult for close others, such as family members (Orford et al., 2010; Orr et al., 2014; Ray et al., 2007; Rodriguez et al., 2014). Living with a relative who excessively drinks or takes drugs is highly stressful (Lindeman et al., 2021; Orford et al., 2010)

Orford (2017, p. 9) points out that although the harm experienced by all family members living with a member’s PSU is similar to an extent, such family harm is variable and depends in important ways on relationship, social, and cultural factors. Orford states that it is essential to keep this variation in family members’ experiences in mind. Lindeman et al. (2021) have produced a summary of the studies exploring the impact of an adult family member’s PSU on family life. Their meta-ethnography states that rather than seeing the consequences for the family members simply as a “problem” or a “difficulty”, the situation can be viewed as an intrusion that overshadows all other aspects of life and is a health risk to all involved family members (Lindeman et al., 2021). The studies refer to families’ endless adaptation to a constantly changing intruder. Every new strategy of adaptation and coping brought hope to the families initially, but such hope soon turned to despair when it became clear that such strategies for adapting were inadequate (Lindeman et al., 2021).

This meta-ethnography focuses on the impact of PSU on families when a substance-using family member is a young family member aged 12–26. Some of the young family members aged 12–26 have already used substances for several years, but all of them are in an early phase of life. The period from adolescence to young adulthood is characterized by numerous developmental transitions, including changes in social roles, and it is a critical period for the development of substance use problems (Cadigan et al., 2019). The pathway from initiation to problematic use of substances among young people is complex and influenced by several factors (World Drug Report, 2018). These factors are both at the personal level (such as behavioural and mental health, neurological developments, and gene variations resulting from social influences), micro-level (parental and family functioning, connectedness to school staff and peers, and friend influences), and macro-level (the socioeconomic and physical environment) (Atherton et al., 2016; Moore et al., 2018; World Drug Report, 2018). Lacking connection to family, school, peers, neighbourhood and community influences adolescents’ psychological well-being and predicts problems such as substance use (Jose et al., 2012). Risk factors such as trauma and childhood adversity, mental health problems, poverty and negative school climate are out of the individual’s control and can make young people vulnerable to substance use (Jose et al., 2012; World Drug Report, 2018).

This meta-ethnography focuses on young family members, whether adolescents or young adults. A young family member is expected to have a different position than an adult one. In most countries, for example, parents usually help their children throughout most of their lives, and the direction of such help tends to remain stable until the parents reach the age range of 70 to 75 (Herlofson & Daatland, 2016). The tasks and responsibilities that adult family members are typically expected to carry out differ from what is expected of young family members.

In this study, family life is understood as a social process that unfolds over time, embracing the everyday life of the family and the daily experiences of relations in the family. The study contributes to what is known about how the substance abuse of family members aged 12 to 26 influences family life. This study aims to integrate and synthesize the research into family members’ experiences of family life when a young family member’s substance use is perceived as problematic. To acquire a more extensive understanding of family experiences and their needs for adapted help and support, the following research question is explored: What is the impact of a young family member’s PSU on family life?

## Methods

### Research design

Noblit and Hare’s (1988) meta-ethnography for interpreting, integrating, and synthesizing qualitative studies has been chosen. We recognize and have reflected on the development of meta-ethnography and critical discussion (Bondas & Hall, 2007a; France et al., 2019b; Bondas & Hall, 2007b; Britten et al., 2017; Thorne, 2017a, 2017b). Booth (2019) describes dual heritage from both systematic reviews and primary qualitative research methodologies. The eMERGe guidelines (France, et al., 2019a) are used to guide the review’s reporting to achieve a transparent and accurate account (Appendix I). In meta-ethnography, the relationship between studies informs the basic analytical decision when translating included qualitative studies into each other. The term “translation“is understood here as taking findings from one study and identifying similar findings in another study, although these may be phrased differently (Noblit & Hare, 1988). What it strives for is a broader and deeper understanding in the form of synthesis through themes and metaphors (Noblit & Hare, 1988). Meta-ethnographies of qualitative studies are helpful for developing both new knowledge and evidence-based practice (Bondas et al., 2017).

### Search strategies and outcomes

Systematic literature searches were conducted by the first (SKL) and second (KBT) authors and an academic librarian, Marianne Nesbjørg Tvedt (MNT). In addition, an academic librarian, Gunhild Austrheim (GA), peer-reviewed the electronic search strategy. A wide search strategy was chosen to find studies with rich descriptions of family life and family relationships, which are complex and multifaceted phenomena. The following databases were considered to be relevant to the topic: CINAHL (EBSCO) (1981-), PsycINFO (Ovid) (1806-), SocINDEX (EBSCO) (1908-), Web of Science (1950-), and the Scandinavian database SveMed+. To increase coverage, the first author (SKL) conducted manual searches in the journals *Journal of Substance Use*, *Substance Use & Misuse*, *Journal of Family Therapy*, *Family Relations*, *Addiction*, and *Nordic Studies on Alcohol and Drugs* and using a Scandinavian digital publishing platform for academic journals and books (Idunn). Back-and-forward reference searches (Sandelowski & Barroso 2007; Cooper et al., 2018) were completed twice, in January 2020 (SKL, KBT) and May 2021 (SKL), who examined the titles of all search results, abstracts, and full texts of original qualitative articles; those considered suitable according to the research objective were included.

PIOS elements were used to define eligibility criteria. PIOS is here an acronym for participants (P), intervention/phenomena (I), outcome (O) and study design (S). The inclusion criteria were based on the research question and related to family, next of kin, parent, child, sibling, and spouse (population); family members living with another family member’s PSU (the phenomenon of interest); and qualitative peer-reviewed empirical studies (type of research). Studies with rich descriptions of family life and family relationships were included, while studies primarily focusing on the impact of PSU on individual family members’ lives and coping without providing a description of family life were excluded. Searches were performed without restriction, and an academic librarian (MNT) performed the search in April 2019 (Appendix II). An update search of the databases was performed in June 2020 (MNT).

**Figure 1.**
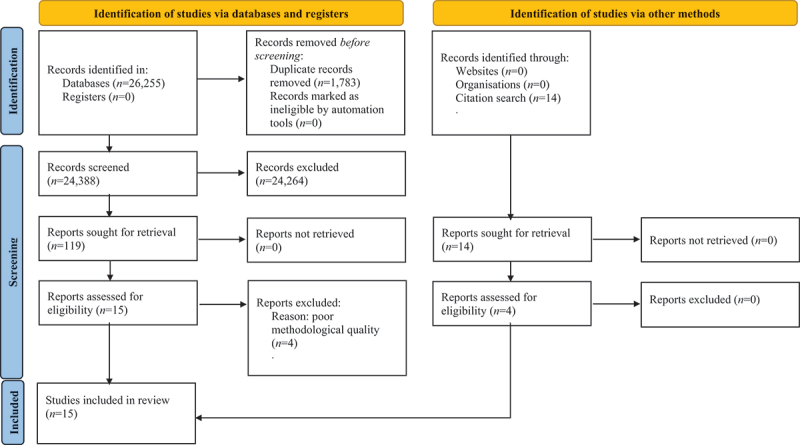
The PRISMA 2020 flow diagram showing the study selection process (see Page et al., 2021).

The systematic search yielded 26,255 records (Figure 1). An additional 14 records were identified in the citation, reference, and journal searches. After reviewing the titles and abstracts and removing duplicates, 24402 studies were assessed against the inclusion and exclusion criteria and 133 studies (119 from databases and 14 from citation search)were subsequently read in full text. At this stage, 114 studies were excluded. During both stages, the entire selection process was executed by SKL and KBT, using the Rayyan application (see Ouzzani et al. (2016) for more information about Rayyan). All full-text articles excluded at this stage of the selection process are presented in the excluded studies table, together with the reason for their exclusion (Appendix III).

### Quality appraisal

The CASP checklist for qualitative research (Critical Appraisal Skills Programme, 2018) was used to critically appraise the studies that met the inclusion criteria (SKL, LL). The overall rating of the studies’ quality was defined according to the risk of bias. We will, in this study, use the term “risk of bias” to indicate the extent to which all aspects of a given study’s credibility, design, and conduct have been evaluated. Questions in both checklists were answered with “Yes”, “Can’t tell”, or “No”. Studies were rated overall as low risk of bias =/< 2 “Can’t tell”; unclear risk of bias>2 “Can’t tell” or “1 No”; high risk of bias =/> “2 No”.

Twelve studies were rated as having a low risk of bias, and three studies as having an unclear risk of bias. Only two studies (Choate, 2015; Smith et al., 2018) adequately addressed the relationship between the researcher and participants. Four studies were rated as being of low methodology quality due to the high risk of bias and were hence excluded (Appendix IV).

### Included studies

The 15 included articles are presented in Table I. Included studies represent the experience of 168 persons: 135 mothers (two of these the mothers of an adopted child), 22 fathers (one the father of an adopted child), five grandmothers, three stepfathers, one stepmother, and two other caretakers. However, some of the studies use the same sample (Groenewald & Bhana, 2016, 2017; Jackson et al., 2007; Mathibela & Skhosana, 2019, 2021; Usher et al., 2007). Studies included in this meta-ethnography represent countries with different welfare systems and substance-use services. Eight studies are from South Africa, three from Australia, two from Canada, and two from Brasil. The young substance-using family members were between the ages of 12 and 26 and used different substances, including cannabis, alcohol, nyaope, and whoonga. Some studies do not differentiate between substances and use terms such as “substances”, “drugs”, “alcohol”, and “psychoactive substances”.

**Table I.** Characteristics of included studies.

First author	Country	Design	Aim/purpose	Sample	Substance	Age of family member with PSU	Results
Asante & Lentoor (2017)	South Africa	Semi-structured interviews	To explore the lived experience of mothers whose children use crystal methamphetamine in a working-class community	*n = 16* mothers	Methamphetamine	15–20 years	Findings show that youth misbehaviour provided a context that led to feelings of shame and embarrassment. Participants also experienced personal challenges, which included emotional problems, fear, and self-blame. Participants also talked about family disruptions and financial drain as adverse experiences resulting from their sons’ misbehaviour.
Choate (2015)	Canada	Semi-structured interviews	To understand how parents have experienced and coped with substance dependence issues that have emerged with their young family members	*n* = 31 17 mothers 7 fathers 1 stepmother 1 stepfather 2 adoptive mothers 1 adoptive father 2 other caregivers	Alcohol and drugs	Teenaged	Findings show that there are unique issues when the person is a young person. Parents need support to be able to see the emerging substance dependence in their young person and how they might effectively respond. They also need support in helping the other children in the family to manage. This work indicates that clinicians should be aware of the need not only to intervene with the identified client but also to create interventions for the entire family system.
Groenewald (2018)	South Africa	Semi-structured interviews	To describe mothers’ experiences of living with an adolescent who is dependent on drugs and to explore the mothers’ accounts of the pernicious behaviours that the adolescents engage(d) in during their drug abuse period	*n=*10 mothers	Drugs	Adolescent	Reveals two common forms of adolescent misconduct that mothers had to contend with, which were “belligerent attitudes and conducts” and “pilfering behaviours”. The mothers further reported that the adolescents’ behaviours negatively affected the mother—adolescent relationship, producing a lack of trust.
Groenewald & Bhana (2017)	South Africa	In-depth interviews facilitated by the lifegrid	To explore the coping responses of mothers whose adolescents have been admitted for treatment for substance abuse.	*n* = 5 mothers	Whoonga, cannabis, and alcohol	15–17 years	Coping emerged as a complex construct in our analysis. The mothers used problem-focused and emotion-focused coping in different combinations of withdrawing, tolerating, and engaged coping responses. The mothers’ coping responses were also influenced by individual and relational factors such as subjective distress and the mother—adolescent relationship.
Groenewald & Bhana (2016)	South Africa	In-depth interviews facilitated by the lifegrid Follow-up interviews	To explore mothers’ experience of living with an adolescent with substance use problems	*n* = 5 mothers	Whoonga, cannabis, and alcohol	15–17 years	The findings illustrate that parenting an adolescent with a substance abuse problem is enormously burdensome for affected mothers. The adolescent’s substance abuse produced several stressful life events, such as adolescent misconduct, family conflict, and financial burden, which were associated with different forms of emotional strain such as hopelessness, guilt, self-blame, worry, shame, anger, and signs of depression.
Jackson & Mannix (2003)	Australia	In-depth interviews	To develop an understanding of the effects of adolescent drug use on family life and gain insight into mothers’ experience of coping with a teen or young adult with a drug problem	*n* = 12 mothers	Cannabis	18–26 years	These findings reveal that when the adolescents developed a drug problem, it was extremely disruptive to the entire family unit.
Jackson et al., (2007)	Australia	Semi-structured interviews	To develop an understanding of the effects of adolescent drug use on family life	*n* = 18 16 mothers 2 fathers	Alcohol and drugs	Adolescent	Findings reveal that the experience of having a drug-abusing adolescent family member had a profound effect on other members of the immediate family. Family relationships were fractured and split as a result of the ongoing destructive and damaging behaviour of the drug-abusing young person.
Kalam & Mthembu (2018)	South Africa	Semi-structured interviews	To explore parents’ experience of parenting adolescents who are abusing substances	*n* = 4 mothers	Drugs	12–18 years	Findings of the current study indicate that authoritarian, authoritative, and permissive parenting styles emerged as strategies that parents had to use in order to manage the situation in their families. Additionally, the findings of this study reveal that adolescent substance abuse has severe consequences for the parent-child subsystem, because it influences parental communication and the family’s wellbeing.
Mathibela & Skhosana (2019)	South Africa	Semi-structured interviews	To obtain an in-depth understanding of the experiences, challenges, and coping strategies of parents raising adolescents abusing substances in the community of Ramotse in Hammanskraal, Gauteng	*n* = 13 9 mothers 4 fathers	Nyaope	13–19 years	It took time for the parents to learn about the abuse of substances by their adolescents. Participants were hardly coping; they survived from day to day. The rights of parents who are raising adolescents that abuse substances are being violated. The South African government does not prioritize the support of parents of adolescents who are abusing substances and this leads to the plight of parents feeling helpless.
Mathibela & Skhosana (2020)	South Africa	Semi-structured interviews	To focus on the coping strategies of parents living with adolescents who are abusing substances	*n* = 13 9 mothers 4 fathers	Nyaope	13–19 years	Parents avoided talking to adolescents to avoid pain and hurt; parents took comfort in their religion by praying or going to church; parents obtained spiritual support from the church and its pastors; parents opted to give money to the adolescents; and, finally, parents said that they still hoped their adolescent child’s behaviour would change.
Smith et al., (2018)	Canada	In-depth interviews	To gain insight into the women’s experience and to explore how they composed themselves as mothers in the midst of prevailing and competing stories of motherhood, family, and substance abuse	*n* = 4 mothers	Alcohol and drugs	Adolescent	Four narrative accounts were composed revealing personal, familial, social, and substance abuse-related complexities in the mothers’ experience. Four narrative threads were also theorized: navigating complexities; loud silences; places, spaces, and the in-between; and living within one another’s stories.
Takahara et al., (2019)	Brazil	Semi-structured interviews	To comprehend the experience of grandmothers who take care of grandchildren who consume psychoactive substances	*n* = 5 grandmothers	Psychoactive substances	Adolescent	The grandmothers recognized that their grandchildren used drugs when their behaviour changed, which forced them to take on the role of counsellor and educator. The ageing process and financial difficulty resulted in limits to care.
Usher et al., (2007)	Australia	In-depth interviews	To describe and construct an interpretation of the lived experience of parenting an adolescent who abuses illicit substances	*n* = 18 16 mothers 2 fathers	Alcohol and drugs	Adolescent	The results indicate that parents struggle to manage the problem, are left to deal with the consequences of the behaviour with little support, and are constantly looking for answers to the questions raised by the problem.
Wegner et al., (2014)	South Africa	Semi-structured interviews	To explore how the occupational performance patterns (roles, rituals, routines, and habits) of mothers were influenced by the addictive behaviours of their drug-dependent young adult children	*n* = 6 mothers	Drugs	18–24 years	Findings provide evidence that the mothers experienced occupational deprivation, imbalance, and alienation from meaningful and purposeful roles, routines, rituals, and habits. As primary caregiver, the mothers were exposed to conditions which required them to adapt so as to deal with the behaviour of their children. This study highlights the need for supportive intervention for such mothers which could address the adaptations required in their occupations as well as promote their wellbeing.
Zerbetto et al.,(2018)	Brazil	Semi-structured family interviews	To discuss the experience of families that care for adolescent consumers of psychoactive substances within the scope of their functioning	*n* = 4 4 family interviews 4 mothers 2 fathers 2 stepfathers	Psychoactive substances	Adolescent	This investigation shows that parents of adolescent consumers of psychoactive drugs, had difficulty establishing an assertive dialogue with their children and developing a hierarchical role, as well as establishing limits. Such situations created ambivalent feelings and negative emotions that mobilized them to search for internal and external resources in relation to the family unit.

### Data analysis and synthesis

Noblit and Hare 1988 describe meta-ethnography as seven phases that overlap. As recommended by Noblit and Hare 1988, the researchers read the included studies repeatedly in order to familiarize themselves with the data.

Data extraction was conducted independently by the first, third, and last authors (SKL, LL, TB) and some issues were discussed in order to obtain a consensus. Next, we analysed the relationship between the studies. Noblit and Hare 1988 state that studies can relate to each other in different ways: they can be compared as analogous (reciprocal translation) or in opposition (refutational translation), or they can be combined into “lines of argument” as aspects of the phenomenon. It was possible to analyse the studies as reciprocal translations, which consist of a lengthy back-and-forth process where the findings of each study are translated into the findings of the other studies. We also analysed whether findings could be understood as conflicting with each other and whether studies accounted for different aspects of the phenomenon (see Noblit and Hare 1988).

A data matrix of each study’s findings was constructed. After several readings and discussions within the team, we determined how the studies were related by juxtaposing the major findings (see Noblit & Hare, 1988). The translation was idiomatic, based on interpretation of meaning, and not literal, i.e., not word for word, and continued until all of the studies were translated. The refutational translation explains differences, exceptions, and inconsistencies across the studies (see Appendix V). Finally, a lines of argument synthesis was created. Based on the themes (5), an overarching metaphor (Metamorphosis) was constructed to express the synthesis (see Noblit and Hare 1988).

The iterative process meant interpreting interpretations of experience. We moved back and forth between the seven phases of meta-ethnography, including the original data, both citations and analysis. The authors’ backgrounds have had a significant influence on this analysis. All of the authors have broad clinical and research experience in health and the social sciences, which has played an essential role in their in-depth and reflective interdisciplinary analysis and subsequent synthesis. The authors represent professions such as family therapist, clinical social worker, and nurse. All of the authors have experience or an interest in family perspectives on substance use and the mental health field. The multicultural, multi-professional, multidisciplinary, and multimethod team collaborated throughout the process under the coordination of the first author. All of the authors contributed to critical and fruitful discussions in repeated virtual meetings on the emerging themes and the overarching metaphor, which was initially suggested by the lead author. Intra- and inter-reviewer negotiations and explications, as well as think-aloud strategies, were helpful and constructive (in accordance with Sandelowski & Barroso, 2007).

## Results

Metamorphosis was adopted as an overarching metaphor based on the findings in the 15 included studies. In all of the included studies, the young family member’s substance use problems were described as causing a colossal upheaval in the family. Family members did not recognize their young family member anymore, and family life changed dramatically. *The Metamorphosis* is one of Franz Kafka’s (2006) best-known works. It tells the story of salesman Gregor Samsa, who wakes one morning to find himself inexplicably transformed into a giant insect and subsequently struggles to adjust to this new condition. The Kafka-inspired metaphor illustrates the extreme change that all the included studies described. Five themes emerged during the translation and thematization of all the included studies: *Stranger in the Family, Injuring Chaos, No Trust Any More, Family Lock-up, and Helpless Societies*. To retain the readability of this article, we have chosen to present the occurrence of the themes in the included articles in Appendix VI and VII. The appendix shows that most themes are presented in all included articles.

### Stranger in the family

The theme *Stranger in the Family* refers to how family members in all the included studies described the beginning of the metamorphosis. Family members were no longer able to see the child they had known before the substance use began. The included studies explained that the change emerged in layers. First, the young family member’s behaviour changed. Subsequently, according to eight studies (Asante & Lentoor, 2017; Choate, 2015; Groenewald, 2018; Smith et al., 2018; Jackson et al., 2007; Takahara et al., 2019; Wegner et al., 2014), anger was the result, while six studies (Groenewald & Bhana, 2016, 2017; Jackson & Mannix, 2003; Kalam et al., 2018; Mathibela & Skhosana, 2019; Usher et al., 2007; Zerbetto et al., 2018) described that young family members attitude changed. As one mother expressed this change in attitudes (Mathibela & Skhosana, 2019, p. 94):

My son was such a respectful child but then he started to be very rude and disrespectful towards the teachers at school then we just knew something was not right. Then I confronted him wanting to understand what was going on.

As a consequence, more distance and anger developed in the family. Eventually, school results and peer connections changed, and many families experienced criminality. Consequently, parents felt that their influence on their young family member had diminished. As one mother described: “Nobody had the right to tell him what he could and couldn’t do and if he wanted to do that in his home that was his business and nobody should be telling him that he can’t do it” (Jackson et al., 2007, p. 327).

The included studies reported that the changes confused other family members for quite some time. It was common for parents not to see the seriousness of the situation until long afterwards and to perceive the first changes as part of normal teenage behaviour. Parents were looking for explanations other than substance use, such as mental health problems, school problems, or past events in family life. As a result, it took a long time for the family to contact substance use services. The exception was when families were confronted with direct evidence, such as overdose, hospitalization, or arrest, and took swift action.

Family members in the included studies responded to young family members’ PSU in a reactive rather than a planned fashion. The included studies described parents trying to regain control by way of confrontations and emotional reactions, such as by crying and through anger. Choate (2015) states that parents who had their own experience of substance use understood the young person’s problems as these related to their own earlier experience, while other parents were confused and struggled to understand.

### Injuring chaos

The theme *Injuring Chaos* reflects the families’ growing desperation, stress, and increased inability to cope effectively. All of the included studies described the families trying almost anything to address their young family member’s PSU. Parents attempted to manage the problems by using various strategies, such as constant vigilance, to control the young family member.

Some parents used strategies that, in retrospect, they saw as “crazy”, as stated by the father and mother in the following (Choate, 2015, p. 468):

For example, a father spoke of confiscating his son’s drugs but then went on to say, “If you have any obligations for what I’ve confiscated, I’ll cover it” (Participant 9, Father). A mother spoke of putting $800 in cash into an envelope so her son could pay off his drug debts that he then headed off to do, wondering “if I would ever see him again” (Participant 21, Mother).

The included studies indicated that family members often felt powerless. Everything they tried failed to make a difference, and nothing seemed to be effective.

### No trust any more

All of the included studies referred to the young family members’ PSU influencing their parents, siblings, and other family members such as grandparents. For many families, substance use-related problems are a multi-generational theme (Choate, 2015; Jackson et al., 2007; Kalam & Mthembu, 2018; Smith et al., 2018; Takahara et al., 2019; Usher et al., 2007; Wegner et al., 2014). Some parents had an upbringing with substance-using parents, some parents had experimented with substance use themselves (Choate, 2015; Smith et al., 2018). In some families, the grandmother had the role of primary carer (Takahara et al., 2019), and in other families the father was absent (Kalam & Mthembu, 2018; Mathibela & Skhosana, 2019, 2021).

The included studies revealed an atmosphere of mistrust and tension between family members. Many of the families experienced terrifying situations, with numerous episodes of violence when the young family member was Under the influence of drugs or were looking for money to buy substances. Family members were constantly afraid. Parents feared their children would be killed or die:

Because I, I didn’t think anything, I can’t think: it’s 12 o’clock he didn’t come home, maybe he is dead, maybe he’s in hospital, maybe he is taken by the police. I think … I can’t think because I’m feeling distracted … maybe he is dead, maybe he’s in hospital, maybe he is taken by the police. (Groenewald & Bhana, 2017, p. 428)

Some of the parents were afraid of being attacked by their children and worried about the safety of their other children:

I am always scared when he needs these drugs because he becomes so violent and disruptive; you can see that he can kill anyone. (Mathibela & Skhosana, 2019, p. 99)

The included studies also describe how the young family member’s PSU has generated family disruption and strained interpersonal relationships within the family. Family members blamed each other or themselves, and their different ideas about how the PSU should be dealt with led to disagreement.

The included studies described siblings often being directly and indirectly affected by their sister’s or brother’s ongoing substance use. Examples of direct effects included being stolen from or assaulted. Some siblings felt so threatened by the drug-dependent sibling that they moved out of the family home (Wegner et al., 2014). The indirect effects included the parents’ focus being solely on the substance-using child. As Choate (2015, p. 470) writes, “In some ways, the siblings lost their brother or sister as well as the family as a unit.”

### Family lock-up

Families who were experiencing the *Metamorphosis* described feelings of severe loneliness and isolation. The theme of *Family Lock-up* shows how family members isolated themselves from close friends, extended family, and the community. The *Family Lock-up* was described as being both self-selected and externally applied. Family members felt unable to seek help or talk to other people about their problems because they felt that others could not understand their situation. They also avoided social engagement and community events because of the criminality of their substance-using family member (Asante & Lentoor, 2017). Some parents also felt isolated from neighbours:

People don’t want to talk to me anymore; others feel that I need to get him arrested, but how do I even do that? He has a case whereby he stole the neighbour’s generator, but the magistrate said he cannot be arrested as he is underage and he was under the influence of drugs, but he should be put under my custody. My neighbour then accused me of bribing the police not to arrest him … I once heard from another neighbour that everyone is talking about me that I protect my son even when he steals from them. The other one told me that they are planning on beating him up if they catch him stealing again because they think I protect him. (Mathibela & Skhosana, 2019, p. 97)

### Helpless societies

The theme of *Helpless Societies* reflects how services, communities, and societies often fail to help families who are living with the PSU of young family members. In countries where the welfare system has fewer resources and where citizens may experience insecurity, families with a young family member from PSU are left alone because help and support, for example from police, child welfare or substance use services, is rarely available, with severe consequences in the form of violence and crime. Still, among all the different cultures represented in the articles, family members seemed to be disappointed by the lack of assistance or the quality of the support provided.

Family members sought help mainly for the young family member with PSU and not for themselves. When they did seek help, the families often found that the help was insufficient or lacking. “No one had anything concrete to tell me, and no one seemed to be able to point me in the right direction” (Smith et al., 2018). The main reason for their lack of assistance is that substance-use services were dependent on the cooperation of the substance-using family member. “Drug rehab couldn’t help because he didn’t want to be helped” (Jackson & Mannix, 2003, p. 173).

Professionals might also offer solutions that conflicted with family values or needs. Parents also had the experience of substance-use services holding back information about their youth’s situation due to confidentiality issues (Choate, 2015). The parents felt disempowered:

It was like the counselor was reprimanding me, and I felt stupid. It is so confusing because when our children are young, we are told to protect them but there is no guidance when they become adolescents and are struggling. When I wasn’t provided the help then I felt hopeless and so ashamed. (Smith et al., 2018, p. 517)

## Discussion

The *Metamorphosis* metaphor reflects the transformative change families were going through when a young family member started and continued to use substances problematically. We have chosen this Kafka-inspired metaphor to emphasize the all-embracing change in the families. In his work *The Metamorphosis*, Kafka (2006) shows Gregor Samsa and his family’s experience of Gregor’s transformation into a giant insect. Kafka wrote that the Samsa family met a hideous fate, worse than any other they knew of. The results in this meta-ethnography show how all-embracing the consequences have been to both the young family member concerned and their families. The change that first transformed the young person continued as an avalanche that also changed family life, health situation and relations. The studies show how the families were “locked up” in the new situation and how inadequate and lacking the support was that they received from others.

In this meta-ethnography, family situations are described mainly from the parents’ perspective; they include the experience of mothers more than that of fathers and lack descriptions of what was experienced by siblings and the substance-using family member. Our findings are in line with those of Orford et al. (2010), who reviewed the experience of family members over the course of two decades of qualitative research. Female partners and mothers were the most represented in their study, and the males who participated were often fathers. Orford (2017, p. 14) also points out that the hardship for family members seems to be more significant in close family relations, particularly those in which the family is characterized by structural subordination with dependence and several burdens. In many of the studies included in this meta-ethnography the mother was the sole provider and had to cope with several practical and economic burdens without either a public or a private safety net. This affects the health situation of women who experience these responsibilities.

The parent’s perspective on the metamorphosis is nevertheless important. In most countries, it is mainly the family that has responsibility for the children (Daatland et al., 2009). The parents’ task is to support their children in their transition to adulthood long after they have reached the age of majority. The included studies show how difficult it was for parents to realize and accept that their child had developed PSU. The parents spent a long time, sometimes several years, trying to find other reasons to explain the changes. When they could no longer use these other explanations, the parents often felt shame and guilt and found that the environment held them responsible. This was a new and highly stressful situation for the parents, one in which they wanted to help their child but did not know how to do so. At the same time, advice from substance use services was lacking or perceived as unhelpful. As a meta-ethnography on adults with PSU (Lindeman et al., 2021) concludes, there are several traits associated with PSU that mean that it places incredible demands on families. Recovery from substance-use problems is a process with an unknown course, as PSU can result in recovery or in life-threatening and/or long-lasting illness. The distinction between the earlier meta-ethnography (Lindeman et al., 2021) and the current study is the impact of time. When a young family member starts using substances problematically, family members are very determined to find help and solutions to the young person’s problems. They are at the beginning of the process, which often means they experience powerlessness and uncertainty over the outcome. With time, the families find ways to survive, which may mean feeling resigned and putting distance between themselves and the substance-using family member (Lindeman et al., 2021). There are also differences in the expectations of family members in terms of different positions, ages, and family roles. In this meta-ethnography, the substance-using family member is young, a child in the family, and family life is described from the parent’s perspective. The responsibility experienced in the relationship between the parent and their young child is different to that in other relationships, such as between adult siblings, of parents towards adult children, of a child towards a parent, or between partners.

The role of the parents in the life of a young substance-using family member requires a complex and nuanced discussion. The World Drug Report (2018) shows that the pathway from initiation to PSU among young people is complex and influenced by several factors. As the World Drug Report (2018) concludes, it is important to keep in mind that it is the critical combination of risk factors that are present and the protective factors that are absent at a particular stage in a young person’s life that make the difference in their susceptibility to drug use. As explained in World Drug Report (2018, p. 6):

Early mental and behavioural health problems, poverty, lack of opportunities, isolation, lack of parental involvement and social support, negative peer influences, and poorly equipped schools are more common among those who develop problems with substance use than among those who do not.

A lack of parental involvement and social support may nevertheless be part of the picture. For many families, PSU is a multi-generation theme, and some family members have a family history of difficult childhood or childhood maltreatment (Zarse et al., 2019). For example, the Adverse Childhood Experience Questionnaire (ACE-Q) provided substantial evidence of the link between adverse childhood experiences and mental and physical illness in adulthood (Felitti et al., 1998; Zarse et al., 2019). In our study, the multi-generational theme shows a family vulnerability, where troubles may have been part of family life for generations. Orford (2017, p. 14) offers an important hypothesis on variation in the accumulated burden for family members. The more that a family member lacks financial or socio-economical resources and the more that the family member faces other hardships, the greater also is the burden of PSU. The greater the accumulated burden that the family member bears, the more challenging it is to cope with a relative’s PSU. As Orford explains the consequences to family members (Orford, 2017, p. 14):

The greater the degree to which an AFM (affected family member) is exposed to family disharmony associated with a relative’s addiction problem, the greater the level of AFM coping difficulty and strain. Family disharmony, or lack of family cohesion, may be a complex concept with multiple indications, but a key index of disharmony is the presence and extent of domestic violence including physical violence, emotional abuse and coercive control.

This is an important wake-up call for substance use services, which still struggle to incorporate family involvement into routine treatment practices in many countries. However, there are interventions already that include family and network perspectives. For example, several systemic family-therapeutic approaches are well-suited to this (see Lorås and Ness (2019)). The 5-Step Method for affected family members is also an acknowledged and research-based method that is suitable for reducing family-related harm from addiction (Copello et al., 2010). The research also shows encouraging results on the effect of family interventions both in PSU patterns and in family functioning (Akram & Copello, 2013). This is also an important reminder of the need for differentiated services and support, where those with a more significant accumulated burden and vulnerability across generations need comprehensive help for their families. Our opinion is that intergenerational problems should not be reduced to individual problems and that it is important to keep the bigger relational picture in mind in health and social services.

This meta-ethnography also suggests how important it is to keep in mind the societal conditions of families. Several included studies are from South Africa. Qualitative studies in Europe, Asia, and the USA appear to be lacking, but the included studies nevertheless represent countries with different political, economic, and cultural situations. When there is a low level of safety and security in society and the society lacks an inclusive welfare system, this exacerbates the lack of protection for both the young substance-using family member and other family members. As a result, families faced crime, threats, and violence alone, without any assistance available to them, as shown in the present study, and for families such as these, homicide related to substance use was a daily threat. Perhaps the geographically varying interest in researching substance-using young people and their families can be linked to the extreme situation families can experience when society lacks an inclusive and easily accessed welfare system.

### Strengths and limitations

A strength of this meta-ethnography is the rigorous methodology of a systematic review with its strengths and opportunities for qualitative synthesis. The meta-ethnography allows the depth and scope to examine participants’ meanings, experiences, and perspectives. Following the eMERGe reporting guidance improves the transparency and wholeness of the research process, which is a quality indicator for meta-ethnography. The flexible methodology of meta-ethnography has allowed us to handle the large number of studies that the search yielded. The systematic, peer-reviewed, and extensive search strategy and the vast number of articles allowed us the opportunity to see different perspectives and ways of describing family lives affected by young family members’ substance use from our own interdisciplinary and multi-professional perspectives, including those derived from our personal experiences. Another strength is the fact that the meta-ethnography team includes experts in health, substance use and family therapy and experts in the meta-ethnography methodology.

The included studies varied in sample size and represented different countries and families. This ensured that there were detailed descriptions of family life and family relationships. However, it is important to keep in mind that the included studies represent parents’ perspectives and other perspectives, for the voices of members of the extended family, of siblings, and of the young family members with PSU are not represented. The female perspective is also more represented because more mothers than fathers are included. There also appears to be a lack of qualitative studies originating in Europe, Asia, and the USA. The studies do not reveal systematic information about the participants’ own problematic substance use or family violence history.

### Implications for future research

The fact that a family member’s PSU affects family life and relations has been documented persuasively by several researchers (Lindeman et al., 2021), especially by Orford and his research group (Copello et al., 2010; Orford et al., 2013; Orford, 2017). We agree with Orford’s (2017) suggestion that, while it is essential to acknowledge cross-cultural similarities in family members’ situations, it is also important to look at the variations and nuances in the experience of family members. Based on the findings of the current meta-ethnography, further research is essential in order to address several critical knowledge gaps. More research is required on the impact on the family during different phases of substance use. It is also essential to include the family members’ PSU perspectives on family life and relationships because this perspective is rarely included in the research. The young person is seen in this study only through the experience of family members, thus more research is needed in order to understand the young person’s situation. As demonstrated by the results of this meta-ethnography, it is vital to include more sibling perspectives, as described both by the siblings themselves and by other family members. It is also important to remember gender perspectives and include the perspectives of fathers, brothers, male partners, and sons. More research is also required within different societies and societal conditions so as to better understand and support families with their accumulated burden. Finally, more research is needed from regions such as Europe, Asia, and the USA, as evidenced by the lack of studies from these regions.

We have included all substances in this study. We think, nevertheless, that it is essential to see the impact of specific substances on family life and understand the differing implications that different substances—ranging from opioids with their high risk of overdose to cannabis, which is legal in some countries, to alcohol and doctor-prescribed medicines—may have for family life.

Family members are often most interested in getting health help for the substance-using family member. More research that focuses on and creates a nuanced understanding of young people’s pathways towards the problematic use of substances and towards recovery processes is important for both families and professionals. We also need in-depth studies of intergenerational substance use problems and how to end a negative family spiral of problematic substance use. We need to know more about how to turn the course of young people’s PSU towards recovery in cooperation with their families.

## Conclusions

The overarching metaphor, the *Metamorphosis*, reflects the all-embracing change experienced by families with an adolescent or young adult family member with PSU. Substance use problems often start at this age and can develop into lifelong chronic health challenges, but they can also lead to recovery. This study shows how powerless and helpless the families often were and how alone and locked up they felt with the *Metamorphosis.*

Family-oriented help must be readily available in this phase of substance use problems. Parents and siblings become deeply involved when a young family member develops substance use. Family members often want to stay involved and provide support but do not know how. Family involvement is often not incorporated into routine treatment practices, and families in crisis are forced to make a big effort to get help.

Kafka (2006, p. 26) wrote that, in the face of the metamorphosis, the Samsa family became so preoccupied with the problems in the present that it lost all ability to move forward. This reminds us of the included studies’ accounts of the loneliness and powerlessness experienced by the family members of a substance-using young person or young adult. We especially hope that our results contribute to an increased awareness of the accumulated burden for some families. Multi-generational and multi-troubled families need extra attention because of their concerning situation. There may be fewer opportunities for them to protect and support not only substance-using young family members but also siblings, the families themselves and forthcoming generations. Another important conclusion is that complex social problems such as PSU require global political attention. The most vulnerable families and family members are often left on their own without support, as the present study indicates, and with the worst consequences of substance use problems, such as terrifying episodes of violence and other horrifying experiences.

## Supplementary Material

Supplemental Material
