# Supplementary material for: The Metamorphosis. The impact of a young family member’s problematic substance use on family life: a meta-ethnography
Source: Int J Qual Stud Health Well-being. 2023 Apr 20;18(1):2202970. doi: 10.1080/17482631.2023.2202970 (PMC10120518; doi:10.1080/17482631.2023.2202970)
Supplement: Supplemental Material [file ZQHW_A_2202970_SM7878.zip › Supplementary files/Appendix I eMERGe reporting guideline.docx]

**Appendix I.** eMERGe Reporting Guidelines

| **Criteria** | **Headings** | **Pages** |
| --- | --- | --- |
| Phase 1  Selecting meta-ethnography  and getting started | 1. Rationale and context for the meta-ethnography  Describe the gap in research or knowledge to be filled by the meta-ethnography and the wider context of the meta-ethnography | 1-3 |
|  | 2. Aim(s) of the meta-ethnography  Describe the meta-ethnography’s aim(s) | 3 |
|  | 3. Focus of the meta-ethnography  Describe the meta-ethnography’s review question(s) (or objectives) | 3 |
|  | 4. Rationale for using meta-ethnography  Explain why meta-ethnography was considered the most appropriate qualitative synthesis methodology | 3 |
| Phase 2  Deciding what is relevant | 5. Search strategy  Describe the rationale for the literature search strategy | 3 |
|  | 6. Search processes  Describe how the literature search was carried out and by whom | 3-4 |
|  | 7. Selection of primary studies  Describe the process of study screening and selection and who was involved | 3-4 |
|  | 8. Outcome of study selection  Describe the results of the study searches and screening | 4-5 |
| Phase 3  Reading included studies | 9. Reading and data-extraction approach  Describe the reading and data-extraction method and processes | 5-6 |
|  | 10. Presenting the characteristics of included studies  Describe the characteristics of the included studies | 5-6 |
| Phase 4  Determining how studies are related | 11. Process for determining how studies are related  Describe the methods and processes for determining how the included studies are related:  - which aspects of the studies were compared?  - how were the studies compared? | 5-6 |
|  | 12. Outcome of relating the studies to each other  Describe how the studies relate to each other | 5-6 |
| Phase 5  Translating studies into one another | 13. Process of translating studies  Describe the methods of translation:  - describe steps taken to preserve the context and meaning of the relationships between concepts within and across studies  - describe how the reciprocal and refutational translations were conducted  - describe how potential alternative interpretations or explanations were considered in the translations | 5-6 |
|  | 14. Outcome of translation  Describe the interpretive findings of the translation | 6-7 |
| Phase 6  Synthesising translations | 15. Synthesis process  Describe the methods used to develop overarching concepts (“synthesised translations”), and describe how potential alternative  interpretations or explanations were considered in the synthesis | 6-7 |
|  | 16. Outcome of synthesis process  Describe the new theory, conceptual framework, model, configuration or interpretation of data developed from the synthesis | 7-15 |
| Phase 7  Expressing the synthesis | 17. Summary of findings  Summarise the main interpretive findings of the translation and synthesis, and compare them to the existing literature | 7-15 |
|  | 18. Strengths and limitations  Reflect on and describe the strengths and limitations of the synthesis:  - methodological aspects: e.g. describe how the synthesis findings were influenced by the nature of the included studies and how the meta-ethnography was conducted | 13 |
|  | 19. Recommendations and conclusions  Describe the implications of the synthesis | 14-15 |
