# Supplementary material for: The Metamorphosis. The impact of a young family member’s problematic substance use on family life: a meta-ethnography
Source: Int J Qual Stud Health Well-being. 2023 Apr 20;18(1):2202970. doi: 10.1080/17482631.2023.2202970 (PMC10120518; doi:10.1080/17482631.2023.2202970)
Supplement: Supplemental Material [file ZQHW_A_2202970_SM7878.zip › Supplementary files/Appendix II Search strategy.docx]

**Appendix II**. Search Strategy

**CINAHL**

The main search was carried out on 24 April 2019.

| **#** | **Query** | **Results** |
| --- | --- | --- |
| S87 | S15 AND S86 | 2,939 |
| S86 | S16 OR S17 OR S18 OR S19 OR S20 OR S21 OR S22 OR S23 OR S24 OR S25 OR S26 OR S27 OR S28 OR S29 OR S30 OR S31 OR S32 OR S33 OR S34 OR S35 OR S36 OR S37 OR S38 OR S39 OR S40 OR S41 OR S42 OR S43 OR S44 OR S45 OR S46 OR S47 OR S48 OR S49 OR S50 OR S51 OR S52 OR S53 OR S54 OR S55 OR S56 OR S57 OR S58 OR S59 OR S60 OR S61 OR S62 OR S63 OR S64 OR S65 OR S66 OR S67 OR S68 OR S69 OR S70 OR S71 OR S72 OR S73 OR S74 OR S75 OR S76 OR S77 OR S78 OR S79 OR S80 OR S81 OR S82 OR S83 OR S84 OR S85 | 886,226 |
| S85 | glaser* | 490 |
| S84 | strauss* n2 corbin* | 327 |
| S83 | corbin* n2 strauss* | 327 |
| S82 | foucault* | 616 |
| S81 | husserl* | 206 |
| S80 | merleau n1 ponty* | 159 |
| S79 | van n1 kaam* | 68 |
| S78 | van n1 manen* | 610 |
| S77 | spiegelberg* | 32 |
| S76 | colaizzi* | 763 |
| S75 | heidegger* | 768 |
| S74 | narrative analys?s | 2,677 |
| S73 | constant n1 comparison | 1,167 |
| S72 | constant n1 comparative | 7,789 |
| S71 | discouse* n3 analys?s | 3 |
| S70 | discouse* n3 analys?s | 3 |
| S69 | discourse* n3 analys?s | 4,809 |
| S68 | content analysis | 37,363 |
| S67 | questionnaire* | 413,495 |
| S66 | observational method* | 21,998 |
| S65 | theme* OR thematic | 90,027 |
| S64 | cluster sampl* | 5,708 |
| S63 | life experience* | 28,831 |
| S62 | lived experience* | 6,510 |
| S61 | life world or life-world or conversation analysis or personal experience* or theoretical saturation | 9,444 |
| S60 | account or accounts or unstructured or open-ended or open ended or text* or narrative* | 136,819 |
| S59 | focus n1 group* | 44,193 |
| S58 | purpos* N4 sampl* | 29,418 |
| S57 | theoretical sampl* | 2,136 |
| S56 | biographical method | 64 |
| S55 | human science | 1,689 |
| S54 | field n1 research | 1,970 |
| S53 | field n1 stud* | 5,439 |
| S52 | humanistic or existential or experimential or paradigm* | 28,247 |
| S51 | action research or cooperative inquir* or co operative inquir* or co-operative inquir* | 8,785 |
| S50 | social construct* OR postmodern* OR post-structural* OR post structural* OR poststructural* OR post modern* OR post-modern* OR feminis* OR interpret* | 90,796 |
| S49 | participant observ* | 11,749 |
| S48 | data n1 saturat* | 662 |
| S47 | emic or etic or hermeneutic$ or heuristic$ or semiotic$ | 5,280 |
| S46 | women’s stor* | 1,080 |
| S45 | life stor* | 1,742 |
| S44 | grounded n1 analys?s | 580 |
| S43 | grounded n1 research | 368 |
| S42 | grounded n1 studies | 1,779 |
| S41 | grounded n1 study | 1,779 |
| S40 | grounded n1 theor* | 16,495 |
| S39 | ethnograph* | 10,864 |
| S38 | ethnonursing | 279 |
| S37 | (MH “Cluster Sample+”) | 4,291 |
| S36 | (MH “Life Experiences+”) | 32,077 |
| S35 | (MH “Phenomenological Research”) | 13,662 |
| S34 | (MH “Phenomenology”) | 3,153 |
| S33 | (MH “Theoretical Sample”) | 1,532 |
| S32 | (MH “Field Studies”) | 2,873 |
| S31 | (MH “Observational Methods+”) | 19,779 |
| S30 | (MH “Purposive Sample”) | 25,960 |
| S29 | (MH “Qualitative Validity+”) | 1,492 |
| S28 | (MH “Constant Comparative Method”) | 6,704 |
| S27 | (MH “Ethnonursing Research”) | 197 |
| S26 | (MH “Ethnological Research”) | 5,903 |
| S25 | (MH “Ethnographic Research”) | 7,007 |
| S24 | (MH “Content Analysis”) | 29,862 |
| S23 | (MH “Discourse Analysis”) | 4,149 |
| S22 | (MH “Focus Groups”) | 37,486 |
| S21 | (MH “Questionnaires”) | 339,642 |
| S20 | (MH “Research, Nursing”) | 19,688 |
| S19 | (MH “Qualitative Studies”) | 94,234 |
| S18 | (MH “Grounded Theory”) | 13,982 |
| S17 | (MH “Audiorecording”) | 39,504 |
| S16 | (MH “Interviews+”) | 192,534 |
| S15 | S9 AND S13 AND S14 | 5,699 |
| S14 | TI ( (family OR families OR parent* OR child* OR daughter* OR son OR sons OR sibling* OR brother* OR sister* OR mother* OR father* OR spouse* OR wife OR wives OR husband* OR partner* OR next of kin OR significant other* OR relative*) N3 (relation* OR dynamic* OR interact* OR impact* OR affect* OR conflict* OR coping OR cope* OR copes OR attitude* OR experience* OR perception* OR perspective* OR burden*) ) OR AB ( (family OR families OR parent* OR child* OR daughter* OR son OR sons OR sibling* OR brother* OR sister* OR mother* OR father* OR spouse* OR wife OR wives OR husband* OR partner* OR next of kin OR significant other* OR relative*) N3 (relation* OR dynamic* OR interact* OR impact* OR affect* OR conflict* OR coping OR cope* OR copes OR attitude* OR experience* OR perception* OR perspective* OR burden*) ) | 101,868 |
| S13 | S10 OR S11 OR S12 | 870,112 |
| S12 | TI ( (family OR families OR parent* OR child* OR daughter* OR son OR sons OR sibling* OR brother* OR sister* OR mother* OR father* OR spouse* OR wife OR wives OR husband* OR partner* OR next of kin OR significant other* OR relative*) ) OR AB ( (family OR families OR parent* OR child* OR daughter* OR son OR sons OR sibling* OR brother* OR sister* OR mother* OR father* OR spouse* OR wife OR wives OR husband* OR partner* OR next of kin OR significant other* OR relative*) ) | 810,537 |
| S11 | (MH “Family Attitudes+”) | 21,895 |
| S10 | (MH “Family+”) | 193,708 |
| S9 | S1 OR S2 OR S3 OR S4 OR S5 OR S6 OR S7 OR S8 | 187,252 |
| S8 | (MH “Substance Abusers+”) | 7,321 |
| S7 | TI alcoholi* OR AB alcoholi* | 10,607 |
| S6 | TI ( drug use* OR drug abuse* OR drug misuse* OR drug overuse* ) OR AB ( drug use* OR drug abuse* OR drug misuse* OR drug overuse* ) | 50,235 |
| S5 | TI ( alcohol use* OR alcohol abuse* OR alcohol misuse* OR alcohol overuse* ) OR AB ( alcohol use* OR alcohol abuse* OR alcohol misuse* OR alcohol overuse* ) | 29,251 |
| S4 | TI ( substance use* OR substance abuse* OR substance misuse* OR substance overuse* ) OR AB ( substance use* OR substance abuse* OR substance misuse* OR substance overuse* ) | 32,106 |
| S3 | TI ( (substance* OR drug* OR alcohol*) N3 (addict* OR dependen* OR habit*) ) OR AB ( (substance* OR drug* OR alcohol*) N3 (addict* OR dependen* OR habit*) ) | 12,868 |
| S2 | (MH “Substance Dependence+”) | 80,879 |
| S1 | (MH “Substance Abuse+”) | 58,005 |

**PsycINFO**

The main search was carried out on 24 April 2019.

| **#** | **Searches** | **Results** |
| --- | --- | --- |
| 1 | exp drug abuse/ | 93,238 |
| 2 | exp Drug Dependency/ | 21,344 |
| 3 | exp Alcohol Abuse/ | 38,925 |
| 4 | ((substance* or drug* or alcohol*) adj3 (addict* or dependen* or habit*)).ab,ti. | 31,179 |
| 5 | (substance use* or substance abuse* or substance misuse* or substance overuse*).ab,ti. | 61,714 |
| 6 | (alcohol use* or alcohol abuse* or alcohol misuse* or alcohol overuse*).ab,ti. | 33,928 |
| 7 | (drug use* or drug abuse* or drug misuse* or drug overuse*).ab,ti. | 41,376 |
| 8 | “alcoholi*”.ab,ti. | 22,581 |
| 9 | 1 or 2 or 3 or 4 or 5 or 6 or 7 or 8 | 167,421 |
| 10 | exp FAMILY/ | 45,268 |
| 11 | exp family relations/ | 98,255 |
| 12 | dysfunctional family/ | 749 |
| 13 | (Family or families or “Next of kin” or “Significant other” or parent* or child* or daughter* or son or sons or sibling* or brother* or sister* or mother* or father* or spouse* or wife or wives or husband* or partner* or relative*).ab,ti. | 1,034,588 |
| 14 | 10 or 11 or 12 or 13 | 1,040,187 |
| 15 | ((Relation* or Dynamic* or Interact* or Impact* or Affect* or Conflict* or Coping or cope or copes or experience* or perception* or perspective* or burden*) adj3 (Family or families or Next of kin or Significant other* or parent* or child* or daughter* or son or sons or sibling* or brother* or sister* or mother* or father* or spouse* or wife or wives or husband* or partner* or relative*)).ab,ti. | 194,437 |
| 16 | 9 and 14 and 15 | 9,596 |
| 17 | interviews/ | 6,510 |
| 18 | exp Grounded Theory/ | 3,407 |
| 19 | exp “Experiences (Events)”/ or exp Life Experiences/ | 52,295 |
| 20 | qualitative research/ | 1 |
| 21 | exp Interviewing/ | 2,574 |
| 22 | exp questionnaires/ | 15,897 |
| 23 | discourse analysis/ | 7,966 |
| 24 | exp Content Analysis/ | 12,447 |
| 25 | exp Ethnography/ | 7,739 |
| 26 | exp NARRATIVES/ | 18,261 |
| 27 | ethnology/ | 441 |
| 28 | exp PHENOMENOLOGY/ | 11,777 |
| 29 | ethnonursing.ab,ti. | 57 |
| 30 | “phenomenol*”.ab,ti. | 35,373 |
| 31 | (grounded adj1 theor*).ab,ti. | 15,604 |
| 32 | (grounded adj1 study).ab,ti. | 205 |
| 33 | (grounded adj1 studies).ab,ti. | 52 |
| 34 | (grounded adj1 research).ab,ti. | 289 |
| 35 | (grounded adj1 analys?s).ab,ti. | 227 |
| 36 | “life stor*”.ab,ti. | 3,158 |
| 37 | “women’s stor*”.ab,ti. | 320 |
| 38 | (emic or etic or hermenutic$ or semiotic$).ab,ti. | 4,480 |
| 39 | (data adj1 saturat*).ab,ti. | 275 |
| 40 | “participant observ*”.ab,ti. | 7767 |
| 41 | (social construct* or postmodern* or post-structural* or post structural* or poststructural* or post modern* or post-modern* or feminis* or interpret*).ab,ti. | 170,958 |
| 42 | (action research or cooperative inquir* or co operative inquir* or co-operative inquir*).ab,ti. | 7,258 |
| 43 | (humanistic or existential or experient* or paradigm*).ab,ti. | 110,758 |
| 44 | (field adj1 stud*).ab,ti. | 6,804 |
| 45 | human science.ab,ti. | 472 |
| 46 | (field adj1 research).ab,ti. | 2,950 |
| 47 | biographical method.ab,ti. | 39 |
| 48 | “theoretical sampl*”.ab,ti. | 519 |
| 49 | (purpos* adj4 sampl*).ab,ti. | 8,728 |
| 50 | (focus adj1 group*).ab,ti. | 32,189 |
| 51 | (account or accounts or unstructured or open-ended or open ended or text* or narrative*).ab,ti. | 287,500 |
| 52 | (life world or life-world or conversation analys?s or personal experience* or theoretical saturation).ab,ti. | 11,671 |
| 53 | “lived experience*”.ab,ti. | 12,370 |
| 54 | “life experience*”.ab,ti. | 8,352 |
| 55 | “cluster sampl*”.ab,ti. | 1,413 |
| 56 | (theme* or thematic).ab,ti. | 112,584 |
| 57 | “observational method*”.ab,ti. | 740 |
| 58 | “questionnaire*”.ab,ti. | 229,085 |
| 59 | content analysis.ab,ti. | 20,593 |
| 60 | (discourse adj3 analys*).ab,ti. | 6,573 |
| 61 | (discurs* adj3 analys*).ab,ti. | 798 |
| 62 | (constant adj1 comparative).ab,ti. | 3,094 |
| 63 | (constant adj1 comparison).ab,ti. | 1,268 |
| 64 | “narrative analys*”.ab,ti. | 2,188 |
| 65 | (heidegger* or colaizzi* or spiegelberg*).ab,ti. | 1,946 |
| 66 | (van adj1 manen*).ab,ti. | 490 |
| 67 | (van adj1 kaam*).ab,ti. | 423 |
| 68 | (merleau adj1 ponty*).ab,ti. | 693 |
| 69 | (husserl* or foucault*).ab,ti. | 3,516 |
| 70 | (corbin* adj2 strauss*).ab,ti. | 618 |
| 71 | (strauss* adj2 corbin*).ab,ti. | 618 |
| 72 | “glaser*”.ab,ti. | 972 |
| 73 | 17 or 18 or 19 or 20 or 21 or 22 or 23 or 24 or 25 or 26 or 27 or 28 or 29 or 30 or 31 or 32 or 33 or 34 or 35 or 36 or 37 or 38 or 39 or 40 or 41 or 42 or 43 or 44 or 45 or 46 or 47 or 48 or 49 or 50 or 51 or 52 or 53 or 54 or 55 or 56 or 57 or 58 or 59 or 60 or 61 or 62 or 63 or 64 or 65 or 66 or 67 or 68 or 69 or 70 or 71 or 72 | 932,575 |
| 74 | 16 and 73 | 2,968 |

**SocINDEX**

The main search was carried out on 28 April 2019.

| S61 | S14 AND S60 | 1,522 |
| --- | --- | --- |
| S60 | S15 OR S16 OR S17 OR S18 OR S19 OR S20 OR S21 OR S22 OR S23 OR S24 OR S25 OR S26 OR S27 OR S28 OR S29 OR S30 OR S31 OR S32 OR S33 OR S34 OR S35 OR S36 OR S37 OR S38 OR S39 OR S40 OR S41 OR S42 OR S43 OR S44 OR S45 OR S46 OR S47 OR S48 OR S49 OR S50 OR S51 OR S52 OR S53 OR S54 OR S55 OR S56 OR S57 OR S58 OR S59 | 392,788 |
| S59 | TI (glaser*) OR AB (glaser*) | 404 |
| S58 | TI (corbin* n2 strauss*) OR AB (corbin* n2 strauss*) | 91 |
| S57 | TI (husserl* or foucault*) OR AB (husserl* OR foucault*) | 4,665 |
| S56 | TI (merleau n1 ponty*) OR AB (merleau n1 ponty*) | 232 |
| S55 | TI (van n1 kaam*) OR AB (van n1 kaam*) | 16 |
| S54 | TI (van n1 manen*) OR AB (van n1 manen*) | 36 |
| S53 | TI (heidegger* or colaizzi* or spiegelberg*) OR AB (heidegger* or colaizzi* or spiegelberg*) | 823 |
| S52 | TI (narrative analys?s) OR AB (narrative analys?s) | 2,136 |
| S51 | TI (constant n1 comparison) OR AB (constant n1 comparison) | 219 |
| S50 | TI (constant n1 comparative) OR AB (constant n1 comparative) | 311 |
| S49 | TI (discurs* n3 analys?s) OR AB (discurs* n3 analys?s) | 600 |
| S48 | TI (discourse* n3 analys?s) OR AB (discourse* n3 analys?s) | 4,142 |
| S47 | TI (content analysis) OR AB (content analysis) | 8,557 |
| S46 | TI (observational method*) OR AB (observational method*) | 427 |
| S45 | TI (theme* or thematic) OR AB (theme* or thematic) | 37,282 |
| S44 | TI (lived experience*) OR AB (lived experience*) | 3,297 |
| S43 | TI (life world or life-world or conversation analys?s or personal experience*) OR AB (life world or life-world or conversation analys?s or personal experience*) | 8,724 |
| S42 | TI (account or accounts or unstructured or opend-ended or open ended or text* or narrative*) OR AB (account or accounts or unstructured or opend-ended or open ended or text* or narrative*) | 128,949 |
| S41 | TI (focus n1 group*) OR AB (focus* n1 group*) | 10,090 |
| S40 | TI (purpos* n4 sampl*) OR AB (purpos* n4 sampl*) | 1,410 |
| S39 | TI (theoretical sampl*) OR AB (theoretical sampl*) | 293 |
| S38 | TI (biographical method) OR AB (biographical method) | 183 |
| S37 | TI (human science) OR AB (human science) | 2,573 |
| S36 | TI (field n1 research) OR AB (field n1 research) | 3,640 |
| S35 | TI (field n1 stud*) OR AB (field n1 stud*) | 4,893 |
| S34 | TI (humanistic or existential or experiential or paradigm*) OR AB (humanistic or existential or experiential or paradigm*) | 27,661 |
| S33 | TI (action research or cooperative inquir* or co operative inquir* or co-operative inquir*) OR AB (action resarch or cooperative inquir* or co operative inquir* or co-operative inquir*) | 41 |
| S32 | TI (social construct* or postmodern* or post-structural* or post structural* or poststructural* or post modern* or post-modern* or feminis* or interpret*) OR AB (social construct* or postmodern* or post-structural* or post structural* or poststructural* or post modern* or post-modern* or feminis* or interpret*) | 47,043 |
| S31 | TI (participant observ*) OR AB (participant observ*) | 5,539 |
| S30 | TI (data n1 saturat*) OR AB (data n1 saturat*) | 24 |
| S29 | TI (emic OR etic or hermeneutic$ OR heuristic$ OR semiotic$) OR AB (emic OR etic or hermeneutic$ OR heuristic$ OR semiotic$) | 6,738 |
| S28 | TI (women’s stor*) OR AB (women’s stor*) | 1,678 |
| S27 | TI (life stor*) OR AB (life stor*) | 2,589 |
| S26 | TI (grounded n1 analys*) OR AB (grounded n1 analys*) | 464 |
| S25 | TI (grounded n1 research) OR AB (grounded n1 research) | 264 |
| S24 | TI (grounded n1 stud*) OR AB (grounded n1 stud*) | 430 |
| S23 | TI (grounded n1 theor*) OR AB (grounded n1 theor*) | 3,426 |
| S22 | TI (phenomenol*) OR AB (phenomenol*) | 5,637 |
| S21 | TI (ethnograph*) OR AB (ethnograph*) | 25,070 |
| S20 | TI (ethnonursing) OR AB (ethnonursing) | 6 |
| S19 | TI (questionnaire*) OR AB (questionnaire*) | 41,946 |
| S18 | TI (qualitative validity) OR AB (qualitative validity*) | 105 |
| S17 | TI (qualitative stud*) OR AB (qualitative stud*) | 13,503 |
| S16 | TI (audiorecord*) or AB (audiorecord*) | 21 |
| S15 | TI (interview*) or AB (interview*) | 100,323 |
| S14 | (TI ( (Relation* or Dynamic* or Interact* or Impact* or Affect* or Conflict* or Coping or cope or copes or attitude* attitude* OR experience* OR perception* OR perspective* OR burden* ) N3 (Family or families or Next of kin or Significant other* or parent* or child* or daughter* or son or sons or sibling* or brother* or sister* or mother* or father* or spouse* or wife or wives or husband* or partner* or relative*) ) OR AB ( (Relation* or Dynamic* or Interact* or Impact* or Affect* or Conflict* or Coping or cope or copes or attitude* attitude* OR experience* OR perception* OR perspective* OR burden* ) N3 (Family or families or Next of kin or Significant other* or parent* or child* or daughter* or son or sons or sibling* or brother* or sister* or mother* or father* or spouse* or wife or wives or husband* or partner* or relative*) )) AND (S9 AND S12 AND S13) | 5,005 |
| S13 | TI ( (Relation* or Dynamic* or Interact* or Impact* or Affect* or Conflict* or Coping or cope or copes or attitude* attitude* OR experience* OR perception* OR perspective* OR burden* ) N3 (Family or families or Next of kin or Significant other* or parent* or child* or daughter* or son or sons or sibling* or brother* or sister* or mother* or father* or spouse* or wife or wives or husband* or partner* or relative*) ) OR AB ( (Relation* or Dynamic* or Interact* or Impact* or Affect* or Conflict* or Coping or cope or copes or attitude* attitude* OR experience* OR perception* OR perspective* OR burden* ) N3 (Family or families or Next of kin or Significant other* or parent* or child* or daughter* or son or sons or sibling* or brother* or sister* or mother* or father* or spouse* or wife or wives or husband* or partner* or relative*) ) | 87,845 |
| S12 | S10 OR S11 | 499,421 |
| S11 | TI ( Family or families or “Next of kin” or “Significant other” or parent* or child* or daughter* or son or sons or sibling* or brother* or sister* or mother* or father* or spouse* or wife or wives or husband* or partner* OR relative* ) OR AB ( Family or families or “Next of kin” or “Significant other” or parent* or child* or daughter* or son or sons or sibling* or brother* or sister* or mother* or father* or spouse* or wife or wives or husband* or partner* OR relative* ) | 497,328 |
| S10 | DE “FAMILIES” OR DE “FAMILIES & psychology” OR DE “FAMILY adaptability” OR DE “FAMILY attitudes” OR DE “FAMILY communication” OR DE “FAMILY conflict” OR DE “FAMILY crises” OR DE “FAMILY health” OR DE “FAMILY relations” OR DE “FAMILY stability” OR DE “FAMILY studies” OR DE “DYSFUNCTIONAL families” | 40,341 |
| S9 | S1 OR S2 OR S3 OR S4 OR S5 OR S6 OR S7 OR S8 | 87,769 |
| S8 | TI alcoholi* OR AB alcoholi* | 13,494 |
| S7 | TI ( alcohol use* or alcohol abuse* or alcohol misuse* or alcohol overuse* ) OR AB ( alcohol use* or alcohol abuse* or alcohol misuse* or alcohol overuse* ) | 20,257 |
| S6 | TI ( drug use* or drug abuse* or drug misuse* or drug overuse* ) OR AB ( drug use* or drug abuse* or drug misuse* or drug overuse* ) | 36,526 |
| S5 | TI ( substance use* or substance abuse* or substance misuse* or substance overuse* ) OR AB ( substance use* or substance abuse* or substance misuse* or substance overuse* ) | 24,202 |
| S4 | TI ( (substance* or drug* or alcohol*) N3 (addict* or dependen*or habit*) ) OR AB ( (substance* or drug* or alcohol*) N3 (addict* or dependen*or habit*) ) | 5,498 |
| S3 | DE “ALCOHOLIC fathers” OR DE “ALCOHOLICS” OR DE “ALCOHOLISM” | 14,163 |
| S2 | DE “DRUG abuse” OR DE “DRUG abusers” OR DE “DRUG addiction” OR DE “DRUG addicts” | 20,552 |
| S1 | DE “SUBSTANCE abuse” | 13,430 |

**SveMed+**

The main search was carried out 28 April 2019.

| 1 | [exp:”substance related disorders”](https://svemedplus.kib.ki.se/Default.aspx?query=exp:%22substance%20related%20disorders%22) | 1,406 |
| --- | --- | --- |
| 2 | [exp:”alcoholics”](https://svemedplus.kib.ki.se/Default.aspx?query=exp:%22alcoholics%22) | 1 |
| 3 | [exp:”drug users”](https://svemedplus.kib.ki.se/Default.aspx?query=exp:%22drug%20users%22) | 62 |
| 5 | [substance overuse](https://svemedplus.kib.ki.se/Default.aspx?query=substance%20overuse) | 1 |
| 6 | [substance abuse](https://svemedplus.kib.ki.se/Default.aspx?query=substance%20abuse) | 3,280 |
| 7 | [substance misuse](https://svemedplus.kib.ki.se/Default.aspx?query=substance%20misuse) | 13 |
| 8 | [substance use](https://svemedplus.kib.ki.se/Default.aspx?query=substance%20use) | 210 |
| 9 | [drug use](https://svemedplus.kib.ki.se/Default.aspx?query=drug%20use) | 748 |
| 10 | [drug abuse](https://svemedplus.kib.ki.se/Default.aspx?query=drug%20abuse) | 3,261 |
| 11 | [drug misuse](https://svemedplus.kib.ki.se/Default.aspx?query=drug%20misuse) | 29 |
| 12 | [drug overuse](https://svemedplus.kib.ki.se/Default.aspx?query=drug%20overuse) | 17 |
| 13 | [alcohol overuse](https://svemedplus.kib.ki.se/Default.aspx?query=alcohol%20overuse) | 0 |
| 14 | [alcohol misuse](https://svemedplus.kib.ki.se/Default.aspx?query=alcohol%20misuse) | 12 |
| 15 | [alcohol abuse](https://svemedplus.kib.ki.se/Default.aspx?query=alcohol%20abuse) | 847 |
| 16 | [alcoholic OR alcoholics OR alcoholism](https://svemedplus.kib.ki.se/Default.aspx?query=alcoholic%20OR%20alcoholics%20OR%20alcoholism) | 2,261 |
| 17 | [alcohol OR drug OR substance](https://svemedplus.kib.ki.se/Default.aspx?query=alcohol%20OR%20drug%20OR%20substance) | 13,118 |
| 18 | [addict OR depend OR habit](https://svemedplus.kib.ki.se/Default.aspx?query=addict%20OR%20depend%20OR%20habit) | 1,292 |
| 19 | [#17 AND #18](https://svemedplus.kib.ki.se/Default.aspx?query=%2317%20AND%20%2318) | 560 |
| 20 | [#1 OR #2 OR #3 OR #5 OR #6 OR #7 OR #8 OR #9 OR #10 OR #11 OR #12 OR #13 OR #14 OR #15 OR #16 OR #19](https://svemedplus.kib.ki.se/Default.aspx?query=%231%20OR%20%232%20OR%20%233%20OR%20%235%20OR%20%236%20OR%20%237%20OR%20%238%20OR%20%239%20OR%20%2310%20OR%20%2311%20OR%20%2312%20OR%20%2313%20OR%20%2314%20OR%20%2315%20OR%20%2316%20OR%20%2319) | 4,641 |
| 21 | [exp:”family”](https://svemedplus.kib.ki.se/Default.aspx?query=exp:%22family%22) | 4,083 |
| 24 | [Family OR families OR “Next of kin” OR “Significant other” OR parent OR child or daughter or son or sibling or brother or sister or mother or father or spouse or wife or wives or husband or partner or relative](https://svemedplus.kib.ki.se/Default.aspx?query=Family%20OR%20families%20OR%20%22Next%20of%20kin%22%20OR%20%22Significant%20other%22%20OR%20parent%20OR%20child%20or%20daughter%20or%20son%20or%20sibling%20or%20brother%20or%20sister%20or%20mother%20or%20father%20or%20spouse%20or%20wife%20or%20wives%20or%20husband%20or%20partner%20or%20relative) | 8,364 |
| 25 | [#21 OR #24](https://svemedplus.kib.ki.se/Default.aspx?query=%2321%20OR%20%2324) | 8,364 |
| 26 | [#20 AND #25](https://svemedplus.kib.ki.se/Default.aspx?query=%2320%20AND%20%2325) | 297 |

**Web of Science - Social Science Index**

The main search was carried out 28 April 2019.The number of results in Web of Science was very high, and a large proportion were not relevant. Therefore, the search was limited to the Social Science Index (i.e. just one of the databases in Web of Science). The search results were subsequently limited to relevant research areas, and results within non-relevant areas were removed (e.g. medicine, pharmacology, infectious diseases).

| # 62 | [11,939](http://apps.webofknowledge.com/summary.do?product=WOS&doc=1&qid=272&SID=F3B4rWrNIPoeUaKdfQH&search_mode=Analyze&update_back2search_link_param=yes) | #58 AND #11  Refined by: [excluding] WEB OF SCIENCE CATEGORIES: ( MEDICINE GENERAL INTERNAL OR HEALTH POLICY SERVICES OR SOCIAL SCIENCES BIOMEDICAL OR CLINICAL NEUROLOGY OR HEALTH CARE SCIENCES SERVICES OR NEUROSCIENCES OR PHARMACOLOGY PHARMACY OR INFECTIOUS DISEASES OR SOCIAL SCIENCES INTERDISCIPLINARY OR OBSTETRICS GYNECOLOGY ) AND [excluding] WEB OF SCIENCE CATEGORIES: ( PEDIATRICS OR GERONTOLOGY OR GERIATRICS GERONTOLOGY OR ENVIRONMENTAL SCIENCES OR NUTRITION DIETETICS OR LAW ) AND [excluding] WEB OF SCIENCE CATEGORIES: ( UROLOGY NEPHROLOGY OR SURGERY OR MEDICAL INFORMATICS OR SPORT SCIENCES OR AUDIOLOGY SPEECH LANGUAGE PATHOLOGY OR HOSPITALITY LEISURE SPORT TOURISM OR OPERATIONS RESEARCH MANAGEMENT SCIENCE OR ORTHOPEDICS OR TRANSPORTATION OR PARASITOLOGY OR EMERGENCY MEDICINE OR BIOTECHNOLOGY APPLIED MICROBIOLOGY OR TROPICAL MEDICINE OR BIOLOGY OR IMMUNOLOGY OR ENVIRONMENTAL STUDIES OR PERIPHERAL VASCULAR DISEASE OR BIOCHEMISTRY MOLECULAR BIOLOGY OR RESPIRATORY SYSTEM OR DENTISTRY ORAL SURGERY MEDICINE OR TOXICOLOGY OR GEOGRAPHY OR AREA STUDIES OR MEDICINE LEGAL OR FOOD SCIENCE TECHNOLOGY OR SOCIAL SCIENCES MATHEMATICAL METHODS OR GASTROENTEROLOGY HEPATOLOGY OR COMPUTER SCIENCE INTERDISCIPLINARY APPLICATIONS OR LINGUISTICS OR LANGUAGE LINGUISTICS OR REHABILITATION OR POLITICAL SCIENCE OR ALLERGY OR CARDIAC CARDIOVASCULAR SYSTEMS OR BUSINESS FINANCE OR INFORMATION SCIENCE LIBRARY SCIENCE OR ENGINEERING CIVIL OR ERGONOMICS OR MATHEMATICAL COMPUTATIONAL BIOLOGY OR HISTORY OR RHEUMATOLOGY OR OTORHINOLARYNGOLOGY OR DERMATOLOGY OR TRANSPLANTATION OR ENDOCRINOLOGY METABOLISM OR ENGINEERING INDUSTRIAL OR HISTORY PHILOSOPHY OF SCIENCE OR MATHEMATICS INTERDISCIPLINARY APPLICATIONS OR ZOOLOGY OR ECONOMICS OR PHYSIOLOGY OR ANESTHESIOLOGY OR CRITICAL CARE MEDICINE OR CELL BIOLOGY OR BUSINESS )  Indexes=SSCI Timespan=All years |
| --- | --- | --- |
| # 61 | [12,611](http://apps.webofknowledge.com/summary.do?product=WOS&doc=1&qid=271&SID=F3B4rWrNIPoeUaKdfQH&search_mode=Analyze&update_back2search_link_param=yes) | #58 AND #11  Refined by: [excluding] WEB OF SCIENCE CATEGORIES: ( MEDICINE GENERAL INTERNAL OR HEALTH POLICY SERVICES OR SOCIAL SCIENCES BIOMEDICAL OR CLINICAL NEUROLOGY OR HEALTH CARE SCIENCES SERVICES OR NEUROSCIENCES OR PHARMACOLOGY PHARMACY OR INFECTIOUS DISEASES OR SOCIAL SCIENCES INTERDISCIPLINARY OR OBSTETRICS GYNECOLOGY ) AND [excluding] WEB OF SCIENCE CATEGORIES: ( PEDIATRICS OR GERONTOLOGY OR GERIATRICS GERONTOLOGY OR ENVIRONMENTAL SCIENCES OR NUTRITION DIETETICS OR LAW )  Indexes=SSCI Timespan=All years |
| # 60 | [13,901](http://apps.webofknowledge.com/summary.do?product=WOS&doc=1&qid=268&SID=F3B4rWrNIPoeUaKdfQH&search_mode=Analyze&update_back2search_link_param=yes) | #58 AND #11  Refined by: [excluding] WEB OF SCIENCE CATEGORIES: ( MEDICINE GENERAL INTERNAL OR HEALTH POLICY SERVICES OR SOCIAL SCIENCES BIOMEDICAL OR CLINICAL NEUROLOGY OR HEALTH CARE SCIENCES SERVICES OR NEUROSCIENCES OR PHARMACOLOGY PHARMACY OR INFECTIOUS DISEASES OR SOCIAL SCIENCES INTERDISCIPLINARY OR OBSTETRICS GYNECOLOGY )  Indexes=SSCI Timespan=All years |
| # 59 | [18,364](http://apps.webofknowledge.com/summary.do?product=WOS&doc=1&qid=267&SID=F3B4rWrNIPoeUaKdfQH&search_mode=CombineSearches&update_back2search_link_param=yes) | #58 AND #11  Indexes=SSCI Timespan=All years |
| # 58 | [1,367,667](http://apps.webofknowledge.com/summary.do?product=WOS&doc=1&qid=266&SID=F3B4rWrNIPoeUaKdfQH&search_mode=CombineSearches&update_back2search_link_param=yes) | #57 OR #56 OR #55 OR #54 OR #53 OR #52 OR #51 OR #50 OR #49 OR #48 OR #47 OR #46 OR #45 OR #44 OR #43 OR #42 OR #41 OR #40 OR #39 OR #38 OR #37 OR #36 OR #35 OR #34 OR #33 OR #32 OR #31 OR #30 OR #29 OR #28 OR #27 OR #26 OR #25 OR #24 OR #23 OR #22 OR #21 OR #20 OR #19 OR #18 OR #17 OR #16 OR #15 OR #14 OR #13 OR #12  Indexes=SSCI Timespan=All years |
| # 57 | [56,449](http://apps.webofknowledge.com/summary.do?product=WOS&doc=1&qid=230&SID=F3B4rWrNIPoeUaKdfQH&search_mode=AdvancedSearch&update_back2search_link_param=yes) | TS=participant observ*  Indexes=SSCI Timespan=All years |
| # 56 | [113,509](http://apps.webofknowledge.com/summary.do?product=WOS&doc=1&qid=231&SID=F3B4rWrNIPoeUaKdfQH&search_mode=AdvancedSearch&update_back2search_link_param=yes) | TS=qualitative stud*  Indexes=SSCI Timespan=All years |
| # 55 | [233](http://apps.webofknowledge.com/summary.do?product=WOS&doc=1&qid=232&SID=F3B4rWrNIPoeUaKdfQH&search_mode=AdvancedSearch&update_back2search_link_param=yes) | TS=audiorecord*  Indexes=SSCI Timespan=All years |
| # 54 | [274,399](http://apps.webofknowledge.com/summary.do?product=WOS&doc=1&qid=233&SID=F3B4rWrNIPoeUaKdfQH&search_mode=AdvancedSearch&update_back2search_link_param=yes) | TS=interview*  Indexes=SSCI Timespan=All years |
| # 53 | [1,294](http://apps.webofknowledge.com/summary.do?product=WOS&doc=1&qid=234&SID=F3B4rWrNIPoeUaKdfQH&search_mode=AdvancedSearch&update_back2search_link_param=yes) | TS=(grounded NEAR/1 analys?s)  Indexes=SSCI Timespan=All years |
| # 52 | [324](http://apps.webofknowledge.com/summary.do?product=WOS&doc=1&qid=235&SID=F3B4rWrNIPoeUaKdfQH&search_mode=AdvancedSearch&update_back2search_link_param=yes) | TS=((van NEAR/1 manen*) OR (van NEAR/1 kaam*) )  Indexes=SSCI Timespan=All years |
| # 51 | [639](http://apps.webofknowledge.com/summary.do?product=WOS&doc=1&qid=236&SID=F3B4rWrNIPoeUaKdfQH&search_mode=AdvancedSearch&update_back2search_link_param=yes) | TS=glaser*  Indexes=SSCI Timespan=All years |
| # 50 | [307](http://apps.webofknowledge.com/summary.do?product=WOS&doc=1&qid=237&SID=F3B4rWrNIPoeUaKdfQH&search_mode=AdvancedSearch&update_back2search_link_param=yes) | TS=(corbin* NEAR/2 strauss*)  Indexes=SSCI Timespan=All years |
| # 49 | [5,952](http://apps.webofknowledge.com/summary.do?product=WOS&doc=1&qid=238&SID=F3B4rWrNIPoeUaKdfQH&search_mode=AdvancedSearch&update_back2search_link_param=yes) | TS=(husserl* OR foucault*)  Indexes=SSCI Timespan=All years |
| # 48 | [581](http://apps.webofknowledge.com/summary.do?product=WOS&doc=1&qid=239&SID=F3B4rWrNIPoeUaKdfQH&search_mode=AdvancedSearch&update_back2search_link_param=yes) | TS=merleau ponty*  Indexes=SSCI Timespan=All years |
| # 47 | [2,253](http://apps.webofknowledge.com/summary.do?product=WOS&doc=1&qid=240&SID=F3B4rWrNIPoeUaKdfQH&search_mode=AdvancedSearch&update_back2search_link_param=yes) | TS=(heidegger* OR colaizzi* OR spiegelberg*)  Indexes=SSCI Timespan=All years |
| # 46 | [18,147](http://apps.webofknowledge.com/summary.do?product=WOS&doc=1&qid=241&SID=F3B4rWrNIPoeUaKdfQH&search_mode=AdvancedSearch&update_back2search_link_param=yes) | TS=narrative analys?s  Indexes=SSCI Timespan=All years |
| # 45 | [1,310](http://apps.webofknowledge.com/summary.do?product=WOS&doc=1&qid=242&SID=F3B4rWrNIPoeUaKdfQH&search_mode=AdvancedSearch&update_back2search_link_param=yes) | TS=(constant NEAR/1 comparison)  Indexes=SSCI Timespan=All years |
| # 44 | [2,312](http://apps.webofknowledge.com/summary.do?product=WOS&doc=1&qid=243&SID=F3B4rWrNIPoeUaKdfQH&search_mode=AdvancedSearch&update_back2search_link_param=yes) | TS=(constant NEAR/1 comparative)  Indexes=SSCI Timespan=All years |
| # 43 | [1,120](http://apps.webofknowledge.com/summary.do?product=WOS&doc=1&qid=244&SID=F3B4rWrNIPoeUaKdfQH&search_mode=AdvancedSearch&update_back2search_link_param=yes) | TS=(discurs* NEAR/3 analys?s )  Indexes=SSCI Timespan=All years |
| # 42 | [10,800](http://apps.webofknowledge.com/summary.do?product=WOS&doc=1&qid=245&SID=F3B4rWrNIPoeUaKdfQH&search_mode=AdvancedSearch&update_back2search_link_param=yes) | TS=(discourse* NEAR/3 analys?s)  Indexes=SSCI Timespan=All years |
| # 41 | [58,840](http://apps.webofknowledge.com/summary.do?product=WOS&doc=1&qid=246&SID=F3B4rWrNIPoeUaKdfQH&search_mode=AdvancedSearch&update_back2search_link_param=yes) | TS=content analysis  Indexes=SSCI Timespan=All years |
| # 40 | [241,941](http://apps.webofknowledge.com/summary.do?product=WOS&doc=1&qid=247&SID=F3B4rWrNIPoeUaKdfQH&search_mode=AdvancedSearch&update_back2search_link_param=yes) | TS=questionnaire*  Indexes=SSCI Timespan=All years |
| # 39 | [14,696](http://apps.webofknowledge.com/summary.do?product=WOS&doc=1&qid=248&SID=F3B4rWrNIPoeUaKdfQH&search_mode=AdvancedSearch&update_back2search_link_param=yes) | TS=observational method*  Indexes=SSCI Timespan=All years |
| # 38 | [87,256](http://apps.webofknowledge.com/summary.do?product=WOS&doc=1&qid=249&SID=F3B4rWrNIPoeUaKdfQH&search_mode=AdvancedSearch&update_back2search_link_param=yes) | TS=(theme* OR thematic)  Indexes=SSCI Timespan=All years |
| # 37 | [13,310](http://apps.webofknowledge.com/summary.do?product=WOS&doc=1&qid=250&SID=F3B4rWrNIPoeUaKdfQH&search_mode=AdvancedSearch&update_back2search_link_param=yes) | TS=cluster sampl*  Indexes=SSCI Timespan=All years |
| # 36 | [89,557](http://apps.webofknowledge.com/summary.do?product=WOS&doc=1&qid=251&SID=F3B4rWrNIPoeUaKdfQH&search_mode=AdvancedSearch&update_back2search_link_param=yes) | TS=(life experience*)  Indexes=SSCI Timespan=All years |
| # 35 | [47,690](http://apps.webofknowledge.com/summary.do?product=WOS&doc=1&qid=252&SID=F3B4rWrNIPoeUaKdfQH&search_mode=AdvancedSearch&update_back2search_link_param=yes) | TS=(lived experience*)  Indexes=SSCI Timespan=All years |
| # 34 | [68,014](http://apps.webofknowledge.com/summary.do?product=WOS&doc=1&qid=253&SID=F3B4rWrNIPoeUaKdfQH&search_mode=AdvancedSearch&update_back2search_link_param=yes) | TS=(life world OR life-world OR conversation analys?s OR personal experience* or theoretical saturation)  Indexes=SSCI Timespan=All years |
| # 33 | [382,945](http://apps.webofknowledge.com/summary.do?product=WOS&doc=1&qid=254&SID=F3B4rWrNIPoeUaKdfQH&search_mode=AdvancedSearch&update_back2search_link_param=yes) | TS=(account OR accounts OR unstructured OR open-ended OR open ended or text* OR narrative*)  Indexes=SSCI Timespan=All years |
| # 32 | [10,752](http://apps.webofknowledge.com/summary.do?product=WOS&doc=1&qid=255&SID=F3B4rWrNIPoeUaKdfQH&search_mode=AdvancedSearch&update_back2search_link_param=yes) | TS=(purpos* NEAR sampl*)  Indexes=SSCI Timespan=All years |
| # 31 | [38,605](http://apps.webofknowledge.com/summary.do?product=WOS&doc=1&qid=256&SID=F3B4rWrNIPoeUaKdfQH&search_mode=AdvancedSearch&update_back2search_link_param=yes) | TS=(focus NEAR/1 group*)  Indexes=SSCI Timespan=All years |
| # 30 | [7,886](http://apps.webofknowledge.com/summary.do?product=WOS&doc=1&qid=257&SID=F3B4rWrNIPoeUaKdfQH&search_mode=AdvancedSearch&update_back2search_link_param=yes) | TS=(purpos* NEAR/4 sampl*)  Indexes=SSCI Timespan=All years |
| # 29 | [16,013](http://apps.webofknowledge.com/summary.do?product=WOS&doc=1&qid=258&SID=F3B4rWrNIPoeUaKdfQH&search_mode=AdvancedSearch&update_back2search_link_param=yes) | TS=theoretical sampl*  Indexes=SSCI Timespan=All years |
| # 28 | [682](http://apps.webofknowledge.com/summary.do?product=WOS&doc=1&qid=259&SID=F3B4rWrNIPoeUaKdfQH&search_mode=AdvancedSearch&update_back2search_link_param=yes) | TS=biographical method  Indexes=SSCI Timespan=All years |
| # 27 | [25,388](http://apps.webofknowledge.com/summary.do?product=WOS&doc=1&qid=260&SID=F3B4rWrNIPoeUaKdfQH&search_mode=AdvancedSearch&update_back2search_link_param=yes) | TS=human science  Indexes=SSCI Timespan=All years |
| # 26 | [8,505](http://apps.webofknowledge.com/summary.do?product=WOS&doc=1&qid=261&SID=F3B4rWrNIPoeUaKdfQH&search_mode=AdvancedSearch&update_back2search_link_param=yes) | TS=(field NEAR/1 research*)  Indexes=SSCI Timespan=All years |
| # 25 | [11,334](http://apps.webofknowledge.com/summary.do?product=WOS&doc=1&qid=263&SID=F3B4rWrNIPoeUaKdfQH&search_mode=AdvancedSearch&update_back2search_link_param=yes) | TS=(field NEAR/1 stud*)  Indexes=SSCI Timespan=All years |
| # 24 | [92,813](http://apps.webofknowledge.com/summary.do?product=WOS&doc=1&qid=262&SID=F3B4rWrNIPoeUaKdfQH&search_mode=AdvancedSearch&update_back2search_link_param=yes) | TS=(humanistic or existential or experiential or paradigm*)  Indexes=SSCI Timespan=All years |
| # 23 | [65](http://apps.webofknowledge.com/summary.do?product=WOS&doc=1&qid=264&SID=F3B4rWrNIPoeUaKdfQH&search_mode=AdvancedSearch&update_back2search_link_param=yes) | TS=(action research OR cooperative inquir* ORco operative inquir* OR co-operative inquir*)  Indexes=SSCI Timespan=All years |
| # 22 | [244,112](http://apps.webofknowledge.com/summary.do?product=WOS&doc=1&qid=265&SID=F3B4rWrNIPoeUaKdfQH&search_mode=AdvancedSearch&update_back2search_link_param=yes) | TS=(social construct* OR postmodern* OR post-structural* OR post structural* OR poststructural* OR post modern* OR post-modern* OR feminis* OR interpret*)  Indexes=SSCI Timespan=All years |
| # 21 | [552](http://apps.webofknowledge.com/summary.do?product=WOS&doc=1&qid=228&SID=F3B4rWrNIPoeUaKdfQH&search_mode=AdvancedSearch&update_back2search_link_param=yes) | TS=(data NEAR/1 saturat*)  Indexes=SSCI Timespan=All years |
| # 20 | [29,480](http://apps.webofknowledge.com/summary.do?product=WOS&doc=1&qid=226&SID=F3B4rWrNIPoeUaKdfQH&search_mode=AdvancedSearch&update_back2search_link_param=yes) | TS=(emic OR etic OR hermeneutic$ OR Heuristic$ OR semiotic$)  Indexes=SSCI Timespan=All years |
| # 19 | [1,759](http://apps.webofknowledge.com/summary.do?product=WOS&doc=1&qid=225&SID=F3B4rWrNIPoeUaKdfQH&search_mode=AdvancedSearch&update_back2search_link_param=yes) | TS=women’s stor*  Indexes=SSCI Timespan=All years |
| # 18 | [11,471](http://apps.webofknowledge.com/summary.do?product=WOS&doc=1&qid=224&SID=F3B4rWrNIPoeUaKdfQH&search_mode=AdvancedSearch&update_back2search_link_param=yes) | TS=life stor*  Indexes=SSCI Timespan=All years |
| # 17 | [920](http://apps.webofknowledge.com/summary.do?product=WOS&doc=1&qid=219&SID=F3B4rWrNIPoeUaKdfQH&search_mode=AdvancedSearch&update_back2search_link_param=yes) | TS=(grounded NEAR/1 research)  Indexes=SSCI Timespan=All years |
| # 16 | [2,001](http://apps.webofknowledge.com/summary.do?product=WOS&doc=1&qid=214&SID=F3B4rWrNIPoeUaKdfQH&search_mode=AdvancedSearch&update_back2search_link_param=yes) | TS=(grounded NEAR/1 (study OR studies))  Indexes=SSCI Timespan=All years |
| # 15 | [13,936](http://apps.webofknowledge.com/summary.do?product=WOS&doc=1&qid=209&SID=F3B4rWrNIPoeUaKdfQH&search_mode=AdvancedSearch&update_back2search_link_param=yes) | TS=(grounded NEAR/1 theor* )  Indexes=SSCI Timespan=All years |
| # 14 | [20,943](http://apps.webofknowledge.com/summary.do?product=WOS&doc=1&qid=227&SID=F3B4rWrNIPoeUaKdfQH&search_mode=AdvancedSearch&update_back2search_link_param=yes) | TS=phenomenol*  Indexes=SSCI Timespan=All years |
| # 13 | [33,548](http://apps.webofknowledge.com/summary.do?product=WOS&doc=1&qid=204&SID=F3B4rWrNIPoeUaKdfQH&search_mode=AdvancedSearch&update_back2search_link_param=yes) | TS=ethnograph*  Indexes=SSCI Timespan=All years |
| # 12 | [51](http://apps.webofknowledge.com/summary.do?product=WOS&doc=1&qid=199&SID=F3B4rWrNIPoeUaKdfQH&search_mode=AdvancedSearch&update_back2search_link_param=yes) | TS=ethnonursing  Indexes=SSCI Timespan=All years |
| # 11 | [39,409](http://apps.webofknowledge.com/summary.do?product=WOS&doc=1&qid=198&SID=F3B4rWrNIPoeUaKdfQH&search_mode=AdvancedSearch&update_back2search_link_param=yes) | #10 AND #9 AND #8  Indexes=SSCI Timespan=All years |
| # 10 | [2,277,185](http://apps.webofknowledge.com/summary.do?product=WOS&doc=1&qid=193&SID=F3B4rWrNIPoeUaKdfQH&search_mode=AdvancedSearch&update_back2search_link_param=yes) | TS=(Relation* or Dynamic* or Interact* or Impact* or Affect* or Conflict* or Coping or cope or copes or experience* or perception* or perspective* or burden*)  Indexes=SSCI Timespan=All years |
| # 9 | [1,154,092](http://apps.webofknowledge.com/summary.do?product=WOS&doc=1&qid=188&SID=F3B4rWrNIPoeUaKdfQH&search_mode=AdvancedSearch&update_back2search_link_param=yes) | TS=(Family or families or “Next of kin” or “Significant other” or parent* or child* or daughter* or son or sons or sibling* or brother* or sister* or mother* or father* or spouse* or wife or wives or husband* or partner* or relative*)  Indexes=SSCI Timespan=All years |
| # 8 | [208,788](http://apps.webofknowledge.com/summary.do?product=WOS&doc=1&qid=183&SID=F3B4rWrNIPoeUaKdfQH&search_mode=AdvancedSearch&update_back2search_link_param=yes) | #7 OR #6 OR #5 OR #4 OR #3 OR #2 OR #1  Indexes=SSCI Timespan=All years |
| # 7 | [30,240](http://apps.webofknowledge.com/summary.do?product=WOS&doc=1&qid=177&SID=F3B4rWrNIPoeUaKdfQH&search_mode=AdvancedSearch&update_back2search_link_param=yes) | TS=(alcohol addict* OR alcohol habit* OR alcohol dependen*)  Indexes=SSCI Timespan=All years |
| # 6 | [33,593](http://apps.webofknowledge.com/summary.do?product=WOS&doc=1&qid=171&SID=F3B4rWrNIPoeUaKdfQH&search_mode=AdvancedSearch&update_back2search_link_param=yes) | TS=(drug addict* OR drug habit* OR drug dependen*)  Indexes=SSCI Timespan=All years |
| # 5 | [25,259](http://apps.webofknowledge.com/summary.do?product=WOS&doc=1&qid=165&SID=F3B4rWrNIPoeUaKdfQH&search_mode=AdvancedSearch&update_back2search_link_param=yes) | TS=(substance addict* OR substance habit* Or substance dependen*)  Indexes=SSCI Timespan=All years |
| # 4 | [25,750](http://apps.webofknowledge.com/summary.do?product=WOS&doc=1&qid=159&SID=F3B4rWrNIPoeUaKdfQH&search_mode=AdvancedSearch&update_back2search_link_param=yes) | TS=alcoholi*  Indexes=SSCI Timespan=All years |
| # 3 | [107,909](http://apps.webofknowledge.com/summary.do?product=WOS&doc=1&qid=153&SID=F3B4rWrNIPoeUaKdfQH&search_mode=AdvancedSearch&update_back2search_link_param=yes) | TS=(drug use* or drug abuse* or drug misuse* or drug overuse*)  Indexes=SSCI Timespan=All years |
| # 2 | [75,391](http://apps.webofknowledge.com/summary.do?product=WOS&doc=1&qid=147&SID=F3B4rWrNIPoeUaKdfQH&search_mode=AdvancedSearch&update_back2search_link_param=yes) | TS=(alcohol use* or alcohol abuse* or alcohol misuse* or alcohol overuse*)  Indexes=SSCI Timespan=All years |
| # 1 | [77,717](http://apps.webofknowledge.com/summary.do?product=WOS&doc=1&qid=141&SID=F3B4rWrNIPoeUaKdfQH&search_mode=AdvancedSearch&update_back2search_link_param=yes) | TS=(substance use* or substance abuse* or substance misuse* or substance overuse*)  Indexes=SSCI Timespan=All years |
