# Supplementary material for: The Metamorphosis. The impact of a young family member’s problematic substance use on family life: a meta-ethnography
Source: Int J Qual Stud Health Well-being. 2023 Apr 20;18(1):2202970. doi: 10.1080/17482631.2023.2202970 (PMC10120518; doi:10.1080/17482631.2023.2202970)
Supplement: Supplemental Material [file ZQHW_A_2202970_SM7878.zip › Supplementary files/Appendix III Excluded studies_clean.docx]

**Appendix III.** Excluded Studies

| **Author** | **Reason for exclusion** |
| --- | --- |
| Askian, Krauss, Baba, Kadir, & Sharghi (2016) | This study essentially looks at and focuses on co-dependence |
| Axelsson (2007)  Backett-Milburn, Wilson, Bancroft, & Cunningham-Burley (2008) | Excluded: this is not a scientific article  Adult as a using family member |
| Barnard, M. (2003)  Barnard, M. (2005) | Age of the using family member  Poor methodological quality |
| Biong & Thylstrup (2016)  Brousseau, N. M., Earnshaw, V. A., Menino, D., Bogart, L. M., Carrano, J., Kelly, J. F., & Levy, S. (2020) | Focuses on bereavement  Focus on treatment effects |
| Butler & Bauld (2005) | Age of a using family member |
| Bönnhoff & Larsen (2014)  Carney, T., Chibambo, V., Ward, C., & Myers, B. (2020) | Focus is mainly on the impact on individual family members’ life  Focus on different risk behaviours |
| Chen, Elisha, Timor, & Ronel (2013) | Treatment context |
| Cho (2008)  Choate, P. W. (2011)  Church, S., Bhatia, U., Velleman, R., Velleman, G., Orford, J., Rane, A., & Nadkarni, A. (2018) | Focus is mainly on impact on individual family members’ life  Poor methodological quality  Age of a using family member |
| Clarfield (2018) | Dissertation |
| Cook (1995) | Published before year 2000 |
| de Freitas Melo & Souza Cavalcante (2019) | Focus is mainly on one paradigm: co-dependency |
| de Moraes Horta, Daspett, Tabosa do Egito, & Stefanini de Macedo (2016) | Age of the using family member |
| Delås (2015) | This study essentially looks at the role of shame in children’s lives |
| Dion (2014) | Poor methodological quality |
| Dobbins (1995)  dos Reis, L. M., Aparecida Sales, C., & de Oliveira, M. L. F. (2017) | Published before the year 2000  Age of the using family member |
| dos Santos & da Silva (2012) | Language |
| Elliston-Gittings (2020) | Dissertation |
| Ervik, Ravndal, & Biong (2019)  Fereidouni, Z., Joolaee, S., Fatemi, N. S., Mirlashari, J., Meshkibaf, M. H., & Orford, J. (2015) | Age of the using family member  Age of the using family member |
| Filizola, Perón, Nascimento, Pavarini, & Filho (2006)  Ferraboli, Noeremberg Guimarães, Kolhs, Bernardi Galli, Noeremberg Guimarães, Fern, & Schneider (2015) | Language  Language |
| Fotopoulou & Parkes (2017) | Age of the using family member |
| Francis, A. A. (2012) | Mixed sample |
| Girsang, Y., Susanti, H., & Panjaitan, R. U. (2019)  Graham (2018)  Hamm (2016) | Age of the using family member  Dissertation  Dissertation |
| Haverfield & Theiss (2014) | Online support forum as a context |
| Haverfield, Theiss, & Leustek (2016)  Healer, W., & Reader, M (2020) | Focus is mainly on the characteristics of communication in families  No method described |
| Hill (2015)  Hodges & Copello (2015)  Hoeck, S., & Van Hal, G. (2012)  Holmila, Itapuisto, & Ilva (2011) | Focus is mainly on parenting and the childcare perspective  Age of the using family member  Age of the using family member  Focus is mainly on parenting and the childcare perspective |
| Howard, K. N., Heston, J., Key, C. M., McCrory, E., Serna-McDonald, C., Smith, K. R., & Hendrick, S. S. (2010)  Incerti, L., Henderson-Wilson, C., & Dunn, M. (2014) | No method described  Age of the using family member |
| Itzick, Segal, & Possick (2019) | Focus is mainly on a specific perspective |
| Johnson (2013) | Age of the using family member |
| Krishnan, Orford, Bradbury, Copello, & Velleman (2001) | Age of the using family member |
| Laboy-Garcia, Cruz-Bermudez, & Sosa-Arrufat (2016) | Focus is on the rehabilitation process |
| Lange & Greif (2011) | Focus is mainly on parenting and a childcare perspective |
| Laudet, Magura, Furst, & Kumar (1999)  Law, L. A. (2020) | Published before the year 2000  Dissertation |
| Leverentz (2007) | Conference paper |
| Maltman, Savic, Manning, Dilkes-Frayne, Carter, & Lubman (2019) | Age of the using family member |
| Marinus, van der Westhuizen, & Alpaslan (2017) | The research focus is on specific geographical context |
| McCann & Lubman (2018a) | Focus is on adaptive strategies |
| McCann & Lubman (2018b) | The focus of this study is to understand AFMs’ stigma experience |
| McCann & Lubman (2018c)  McCann, T. V., Polacsek, M., & Lubman, D. I. (2019) | Focus is on barriers to seeking support  Age of the using family member |
| McDonagh, Connolly, & Devaney (2019)  Mechling, B. M., Ahern, N. R., & Palumbo, R. (2018) | Focus is on barriers to seeking support  Age of the using family member |
| Mir, Simpson, de Azevedo, & da Silva Costa (2006) | Language |
| Montgomery & Johnson (1992) | Published before 2000 |
| Moran (1992) | Published before 2000 |
| Morgan & Brosi (2007)  Moriarty, H., Stubbe, M., Bradford, S., Tapper, S., & Lim, B. T. (2011) | Specific focus on an individual family member’s life  Age of the using family member |
| Murray (1998)  Najor, L. A. (2017) | Published before 2000  Dissertation |
| Natera, Mora, & Tiburcio (2001) | Language |
| Naylor & Lee (2011)  Ngantweni, A. (2018) | Mainly specific focus on one type of family dynamics  Dissertation |
| Nimtz, Fornalski Tavares, Alves Maftum, Zerwes Ferreira, de Oliveira Borba, Capistrano, & Carolina (2014) | Treatment context |
| Nordgren, Richert, Svensson, & Johnson (2019)  Näsman & Alexanderson (2017) | Age of the using family member  Age of the using family member |
| Okeke-Agiriga (2016)  Ólafsdóttir, J., Orjasniemi, T., & Hrafnsdóttir, S. (2020) | Dissertation  Age of the using family member |
| Olex (2019) | Dissertation |
| Orford, Natera, Davies, Nava, Mora, Rigby, Bradbury, Copello, & Velleman (1998) | Published before the year 2000 |
| Orford, Rigby, Miller, Tod, Bennett, & Velleman (1992) | Published before the year 2000 |
| Orjasniemi & Kurvinen (2017) | Language |
| O’Shay-Wallace (2019) | The focus of the study is on understanding AFMs’ stigma experience |
| Pandini, Ferreira D’Artibal, Paiano, & Marcon (2016) | Age of the using family member |
| Park & Schepp (2017) | Focus is on the patterns of adaptation |
| Park, Schepp, & Park (2016) | Focus is mainly on the impact on an individual family member’s life |
| Peled & Sacks (2008) | Focus is mainly on the impact on an individual family member’s life |
| Ramirez (2016) | Dissertation |
| Reid | Focus is mainly on the impact on an individual family member’s life |
| Reinaldo & Pillon (2008)  Rhodes (2016) | Focus on case management  Dissertation |
| Richter, Chatterji, & Pierce (2000) | Age of the using family member |
| Ripke (2003) | Dissertation |
| Robertson (2009) | Dissertation |
| Ronel & Haimoff-Ayali (2010) | Focus is mainly on impact on an individual family member’s life |
| Rossato & Kirchhof (2006) | Language |
| Rydelius (2006) | A theoretical article |
| Santos & Martin (2009) | Treatment context |
| Sarpavaara (2014)  Schultz, P., & Alpaslan, A. H. N. (2016) | Focuses specifically on motivational interviewing  Age of the using family member |
| Schäfer (2011)  Seleghim, M. R., & Frari Galera, S. A. (2019) | Treatment context  Age of the using family member |
| de Siqueira, Moreschi, Terra, Soccol, & de Souza Mostardeiro (2015) | Focus on families understanding of the use of substances |
| Simonen & Torronen (2017) | The focus is mainly on individual family members life |
| Smith (2020) | The focus is especially on individual family members life |
| Soccol, Terra, Ribeiro, Teixeira, Siqueira, & Mostardeiro (2014) | Age of the using family member |
| Sundfaer (2005)  Tamutienė & Jogaitė (2019)  Tamutienė and Laslett (2017) | Focus is on specific dynamics in the family  Age of the using family member  Age of the using family member |
| Taylor, Coall, Marquis, & Batten (2016) | Focus is on specific strategies |
| Tedgård, Råstam, & Wirtberg (2019) | Focus mainly on the parenting role |
| Templeton (2012) | This study essentially looks at the role of grandparents |
| Templeton, Velleman, Hardy, & Boon (2009) | Focus is mainly on parenting and the childcare perspective |
| Thorne (2005)  Tinnfalt, A., Froding, K., Larsson, M., & Dalal, K. (2018) | Dissertation  Age of the using family member |
| Wangensteen, Bramness, & Halsa (2019)  Webber, Ruth (2016) | Focus is mainly on specific family relations and individual members  Poor methodological quality |
| Wilson, Lubman, Rodda, Manning, & Yap (2018)  Vind, L. (2010)  Wiseman (1975) | Context of the online counselling sessions  Excluded: this is not a scientific article  Published before the year 2000 |
| Wiseman (1980) | Published before the year 2000 |
| Zajdow (1995) | Published before the year 2000 |
| Zerbetto, Motz Cid, de Souza Gonçalves, & Oliveira Ruiz (2018) | Focus is on specific dynamics in the family |
