# Supplementary material for: The Metamorphosis. The impact of a young family member’s problematic substance use on family life: a meta-ethnography
Source: Int J Qual Stud Health Well-being. 2023 Apr 20;18(1):2202970. doi: 10.1080/17482631.2023.2202970 (PMC10120518; doi:10.1080/17482631.2023.2202970)
Supplement: Supplemental Material [file ZQHW_A_2202970_SM7878.zip › Supplementary files/Appendix V Translation .docx]

| **First author** | **Asante (2017)** | **Choate, (2015)** | **Groenevald (2016)** | **Mathibela (2019)** | **Takahara (2019)** | **Groenewald (2018)** | **Jackson (2003)** | **Jackson, (2007)** | **Kalam (2018)** | **Mathibela (2020)** | **Smith (2018)** | **Wegner (2014)** | **Zerbetto (2018)** | **Usher (2007)** |
| --- | --- | --- | --- | --- | --- | --- | --- | --- | --- | --- | --- | --- | --- | --- |
| Influence the lifestyle of the parent | Son’s misbehaviour, adverse behaviours of the child might influence the lifestyle of the parent | Parents saw their lives beginning to fall into a pattern of chaos. | Mothers referred to the impact of the adolescents’ substance abuse on  their work performance. | Parents felt that their lives revolved around their adolescents who abuse substances, as they cannot plan anything the way they abused to. | Suffered due to the disappearance of her grandsons for days or weeks, without giving explanations. | Expecting trouble when socialize  Searched for her son for 2 days  Concerted efforts to get her son in a local program | Participants reported feeling tied to the house  in much the same way as they had when their  children were much younger. | Exhausted by the demands of being full time careers to the child of their drug abusing child | Quality of life being at risk | Lives being centered on their adolescents  Their lives were trapped  Wanted to be away from home  . | I felt trapped in the fear and carrying the responsibility of my  son’s addiction and mental health. | Time consuming and demanding  Affecting my work | Feelings of fear, frustration, failure, and impotence were stressed out by the parents | The adolescent’s substance use impacted on the  health of the parents as well as their relationship with  each other and other members of the family. |
