# Supplementary material for: The Metamorphosis. The impact of a young family member’s problematic substance use on family life: a meta-ethnography
Source: Int J Qual Stud Health Well-being. 2023 Apr 20;18(1):2202970. doi: 10.1080/17482631.2023.2202970 (PMC10120518; doi:10.1080/17482631.2023.2202970)
Supplement: Supplemental Material [file ZQHW_A_2202970_SM7878.zip › Supplementary files/Appendix VI The analysis process.docx]

**Appendix VI**. The analysis processes.

| **Theme** | **Overview of preliminary thematic groups the theme is based on, and in number of studies** | **The studies the theme is presented in** |
| --- | --- | --- |
| **The stranger in the family** | The changing relation (10 studies)  New peers and families that supported drug use (3 studies)  Criminality/stealing (13 studies)  Change emerged in layers, in a non-linear way (4 studies)  Direct evidence (2 studies)  Not seeing the seriousness (4 studies)  Changes in their youth (15 studies)  Changes as part of the normal teenage years (2 studies)  Reactive rather than planned responses (1 study)  Emotions took control (1 study)  Trying to regain control (7 studies)  Confused for quite some time (4 studies)  Peer connections changed (4 studies)  Stopped seeing the child that they had known before the substance use (2 studies)  ‘He’s got an anger issue in him’ (6 studies)  Diminished influence on their youth (5 studies)  Looking for explanations other than substance (6 studies)  Historical concerns as a basis for understanding (2 studies)  Parents saw the issues of their youth through their own past experiences (1 study)  Without own substance use history reported being confused and struggled to understand (1 study) | Asante & Lentoor (2017)  Choate (2015)  Groenewald (2018)  Groenewald & Bhana (2017)  Groenewald & Bhana (2016)  Jackson & Mannix (2003)  Jackson et al. (2007)  Kalam & Mthembu (2018)  Mathibela & Skhosana (2019)  Mathibela & Skhosana (2020)  Smith et al. (2018)  Takahara et al. (2019)  Usher et al. (2007)  Wegner et al. (2014)  Zerbetto et al. (2018) |
| **Injuring chaos** | Growing desperation, stress and an increased inability to effectively cope (4 studies)  Using “crazy” strategies (2 studies)  Coping by controlling (6 studies)  Confrontation (7 studies)  Powerless and that nothing they were trying seemed effective (5 studies)  Trying almost anything to find a solution (5 study)  Failed to make a difference, no constructive change (5 studies)  Stances (Realizations) (6 studies)  Tolerate or not tolerate (3 studies)  Nothing left to try (5 studies)  Vigilance and ongoing drug testing (3 studies)  Combination of coping responses or changing (4 studies)  Aggressive and controlling form of engaged coping (1 study)  Ignored the adolescent and withdrew from engaging (1 study)  Coping influences (1 study) | Asante & Lentoor (2017)  Choate (2015)  Groenewald (2018)  Groenewald & Bhana (2017)  Groenewald & Bhana (2016)  Jackson & Mannix (2003)  Jackson et al. (2007)  Kalam & Mthembu (2018)  Mathibela & Skhosana (2020)  Smith et al. (2018)  Takahara et al. (2019)  Usher et al. (2007)  Wegner et al. (2014)  Zerbetto et al. (2018) |
| **No trust anymore** | Influence the lifestyle of the parent and family (15 studies)  Psychosocial emotional problems (12 studies)  Feeling hopelessness (6 studies)  Feared that their children could be killed/die (11 studies)  Fear of attack by their own children (9 studies)  Fear of bad news (2 studies)  Fear of the safety of other children (3 studies)  Parents self-blame: did the parent overprotect, spoil, failing as a mother (11 studies)  Youth blaming parents (3 studies)  Family disruptions family chaos - affecting spouse, the children (12 studies)  Lacking co-parent (3 studies)  Parent – sibling relationships (7)  My spouse and I are constantly fighting because he/she does not understand (10 studies)  Financial drain (9 studies)  Multi-generation theme (7 studies) | Asante & Lentoor (2017)  Choate (2015)  Groenewald (2018)  Groenewald & Bhana (2017)  Groenewald & Bhana (2016)  Jackson & Mannix (2003)  Jackson et al. (2007)  Kalam & Mthembu (2018)  Mathibela & Skhosana (2019)  Mathibela & Skhosana (2020)  Smith et al. (2018)  Takahara et al. (2019)  Usher et al. (2007)  Wegner et al. (2014)  Zerbetto et al. (2018) |
| **The lock up** | Inability to seek help or talk to other people about their problems (2 studies)  Coping by withdrawing/ Isolate themselves (11 studies)  Shame and guilt embarrassment (10 studies)  The strained interpersonal relationships community members due to addiction (6 studies) | Asante & Lentoor (2017)  Choate (2015)  Groenewald & Bhana (2017)  Groenewald & Bhana (2016)  Jackson & Mannix (2003)  Jackson et al. (2007)  Mathibela & Skhosana (2019)  Mathibela & Skhosana (2020)  Smith et al. (2018)  Takahara et al. (2019)  Usher et al. (2007)  Wegner et al. (2014)  Zerbetto et al. (2018) |
| **Helpless societies** | Professionals supporting the view that the problems were based in mental health (2 studies)  Positive own help-seeking encouraged her to seek help for her youth (1 study)  Reach out for help (6 studies)  Prof. reluctant to use terms like dependency and addiction (2 studies)  Professionals offered solutions, which were at odds with family values or needs (1 study)  Prof. frequently held back information (2 studies)  Parents disempowered (4 studies)  Prof help not helping (3 studies)  Religion as comfort (1 study)  Youth often just refused to get help (2 studies)  Lack of formal support (7 studies)  Own need for self-care (2 studies)  Parents also found help through their own peers (7 studies)  Social problems (5 studies) | Asante & Lentoor (2017)  Choate (2015)  Groenewald (2018)  Groenewald & Bhana (2017)  Groenewald & Bhana (2016)  Jackson & Mannix (2003)  Jackson et al. (2007)  Kalam & Mthembu (2018)  Mathibela & Skhosana (2019)  Mathibela & Skhosana (2020)  Smith et al. (2018)  Takahara et al. (2019)  Usher et al. (2007)  Wegner et al. (2014)  Zerbetto et al. (2018) |
