# Supplementary material for: The Metamorphosis. The impact of a young family member’s problematic substance use on family life: a meta-ethnography
Source: Int J Qual Stud Health Well-being. 2023 Apr 20;18(1):2202970. doi: 10.1080/17482631.2023.2202970 (PMC10120518; doi:10.1080/17482631.2023.2202970)
Supplement: Supplemental Material [file ZQHW_A_2202970_SM7878.zip › Supplementary files/Appendix VII Studies Contributing to the Review Findings.docx]

| **Subthemes** | **Asante (2017)** | **Choate (2015)** | **Groenewald (2016)** | **Groenewald (2017)** | **Groenewald (2018)** | **Jackson (2003)** | **Jackson (2007)** | **Kalam (2018)** | **Mathibela (2019)** | **Mathibela (2020)** | **Smith (2018)** | **Takahara (2019)** | **Usher (2007)** | **Wegner (2014)** | **Zerbetto (2018)** |
| --- | --- | --- | --- | --- | --- | --- | --- | --- | --- | --- | --- | --- | --- | --- | --- |
| The stranger in the family | X | X | X | X | X | X | X | X | X | X | X | X | X | X | X |
| Injuring chaos | X | X | X | X | X | X | X | X | X | X | X | X | X | X | X |
| No trust anymore | X | X | X | X | X | X | X | X | X | X | X | X | X | X | X |
| The lock-up | X | X | X | X | X | X | X | X | X | X | X | X | X | X | X |
| Helpless societies | X | X | X | X | X | X | X | X | X | X | X | X | X | X | X |
