# Supplementary material for: The Metamorphosis. The impact of a young family member’s problematic substance use on family life: a meta-ethnography
Source: Int J Qual Stud Health Well-being. 2023 Apr 20;18(1):2202970. doi: 10.1080/17482631.2023.2202970 (PMC10120518; doi:10.1080/17482631.2023.2202970)
Supplement: Supplemental Material [file ZQHW_A_2202970_SM7878.zip › Supplementary files/Apppendix IV Critical appraisal_.docx]

| Appendix IV. Critical appraisal. | | | | | | | | | | | |
| --- | --- | --- | --- | --- | --- | --- | --- | --- | --- | --- | --- |
|  | 1. Was there a clear statement of the aims of the research? | 2. Is a qualitative methodology appropriate? | 3. Was the research design appropriate to address the aims of the research? | 4. Was the recruitment strategy appropriate to the aims of the research? | 5. Was the data collected in a way that addressed the research issue? | 6. Has the relationship between researcher and participants been adequately considered? | 7. Have ethical issues been taken into consideration? | 8. Was the data analysis sufficiently rigorous? | 9. Is there a clear statement of findings? | 10. How valuable is the research? | Rating |
| 1. Asante & Lentoor, 2017 | yes | yes | yes | yes | yes | Can’t tell | Yes | yes | yes | Valuable and necessary | Low Risk of Bias |
| 1. Barnard, M. (2005) | yes | yes | Yes | Can’t tell | Can’t tell | Can’t tell | Can’t tell | Can’t tell | yes | Valuable | High Risk of Bias |
| 1. Choate (2015). | yes | yes | Can’t tell | yes | yes | yes | yes | Nei | yes | Valuable and necessary | Unclear Risk of Bias |
| 1. Choate, P. W. (2011). | yes | yes | yes | Can’t tell | Can’t tell | Can’t tell | no | Can’t tell | Can’t tell | Valuable | High Risk of Bias |
| 1. Groenewald (2018) | yes | yes | yes | yes | yes | Can’t tell | Can’t tell | yes | yes | Valuable and necessary | Low Risk of Bias |
| 1. Groenewald, C., & Bhana, A. (2017). | yes | yes | yes | yes | yes | Can’t tell | yes | yes | yes | Valuable  and necessary | Low Risk of Bias |
| 1. Groenewald, C., & Bhana, A. (2016). | yes | yes | yes | yes | yes | Can’t tell | yes | yes | yes | Valuable  and necessary | Low Risk of Bias |
| 1. Jackson, D. and Mannix (2003) | yes | yes | yes | yes | yes | Can’t tell | yes | Can’t tell | yes | Valuable and necessary | Low Risk of Bias |
| 1. Jackson, D., Usher, and O'Brien (2007) | yes | yes | yes | Can’t tell | yes | Can’t tell | yes | Can’t tell | yes | Valuable and necessary | Unclear Risk of Bias |
| 1. Kalam, A., & Mthembu, T. G. (2018). | yes | yes | yes | Yes | Can’t tell | Can’t tell | yes | yes | yes | Valuable and necessary | Low Risk of Bias |
| 1. Mafa and Makhubele (2019) | yes | yes | Can’t tell | Can’t tell | Can’t tell | Can’t tell | yes | no | yes | Valuable | High Risk of Bias |
| 1. Mathibela and Skhosana (2019) | yes | yes | yes | yes | yes | Can’t tell | yes | yes | yes | Valuable and necessary | Low Risk of Bias |
| 1. Mathibela, F., & Skhosana, R. M. (2020). | yes | yes | yes | yes | yes | Can’t tell | yes | Can’t tell | yes | Valuable  and necessary | Low Risk of Bias |
| 1. Smith, J. M., Estefan, A., & Caine, V. (2018). | yes | yes | yes | yes | yes | yes | Can’t tell | yes | yes | Valuable and necessary | Low Risk of Bias |
| 1. Takahara, A. H., Galera, S. A. F., Zanetti, A. C. G., Gonçalves, A. M. D. S., Protti-Zanatta, S. T., & Zerbetto, S. R. (2019). | yes | yes | yes | yes | yes | Can’t tell | yes | yes | yes | Valuable  and necessary | Low Risk of Bias |
| 1. Usher, K., Yesckson, D., & O'Brien, L. (2007). | yes | yes | yes | Can’t tell | yes | Can’t tell | yes | yes | yes | Valuable and necessary | Low Risk of Bias |
| 1. Webber, Ruth (2016) | yes | yes | yes | Can’t tell | Can’t tell | Can’t tell | no | no | yes | Valuable | High Risk of Bias |
| 1. Wegner, Arend, Bassadien, Bismath, and Cros (2014) | yes | yes | yes | yes | Can’t tell | Can’t tell | yes | Can’t tell | yes | Valuable and necessary | Unclear Risk of Bias |
| 1. Zerbetto, S. R., Ruiz, B. O., Galera, S. A. F., & Zanetti, A. C. G. (2018). | yes | yes | yes | yes | yes | Can’t tell | yes | yes | yes | Valuable and necessary | Low Risk of Bias |
